# Overexpression of protein kinase C *ɛ* improves retention and survival of transplanted mesenchymal stem cells in rat acute myocardial infarction

**DOI:** 10.1038/cddis.2015.417

**Published:** 2016-01-21

**Authors:** H He, Z-H Zhao, F-S Han, X-H Liu, R Wang, Y-J Zeng

**Affiliations:** 1 Department of Emergency Cardiology, Beijing Anzhen Hospital, Capital Medical University, Beijing, China; 2 Department of Cardiology, PuDong New Area District ZhouPu Hospital, Shanghai, China; 3 Department of Cardiology, Beijing Anzhen Hospital, Capital Medical University, Beijing, China

## Abstract

We assessed the effects of protein kinase C *ɛ* (PKC*ɛ*) for improving stem cell therapy for acute myocardial infarction (AMI). Primary mesenchymal stem cells (MSCs) were harvested from rat bone marrow. PKC*ɛ*-overexpressed MSCs and control MSCs were transplanted into infarct border zones in a rat AMI model. MSCs and PKC*ɛ* distribution and expression of principal proteins involved in PKC*ɛ* signaling through the stromal cell-derived factor 1 (SDF-1)/CXC chemokine receptor type 4 (CXCR4) axis and the phosphatidylinositol 3 kinase (PI3K)/protein kinase B (AKT) pathway were analyzed by immunofluorescence and western blot 1 day after transplantation. Echocardiographic measurements and histologic studies were performed at 4 weeks after transplantation, and MSC survival, expression of vascular endothelial growth factor (VEGF), basic fibroblast growth factor (bFGF), transforming growth factor *β* (TGF*β*), cardiac troponin I (cTnI), von Willebrand factor (vWF), smooth muscle actin (SMA) and factor VIII and apoptosis in infarct border zones were assessed. Rat heart muscles retained more MSCs and SDF-1, CXCR4, PI3K and phosphorylated AKT increased with PKC*ɛ* overexpression 1 day after transplantation. MSC survival and VEGF, bFGF, TGF*β*, cTnI, vWF, SMA and factor VIII expression increased in animals with PKC*ɛ*-overexpressed MSCs at 4 weeks after transplantation and cardiac dysfunction and remodeling improved. Infarct size and apoptosis decreased as well. Inhibitory actions of CXCR4 or PI3K partly attenuated the effects of PKC*ɛ*. Activation of PKC*ɛ* may improve retention, survival and differentiation of transplanted MSCs in myocardia. Augmentation of PKC*ɛ* expression may enhance the therapeutic effects of stem cell therapy for AMI.

Irreversible and widespread loss of myocardial cells and subsequent ventricular remodeling induced by acute myocardial infarction (AMI) is the main cause of chronic heart failure^1^ and globally >17 million people died of ischemic heart diseases in 2008.^2^ Stem cell-based regenerative therapy for AMI is encouraging with respect to preclinical^3, 4^ and clinical data,^5, 6, 7, 8^ and this may soon be a therapeutic modality for injury resulting from coronary artery disease. Two problems – poor homing of transplanted cells to injury sites, and poor cell survival – require resolution before transplantation therapy can be broadly effective. The stromal cell-derived factor 1 (SDF-1)/CXC chemokine receptor type 4 (CXCR4) axis has an important role during migration, proliferation and survival of stem cells, but using this knowledge to improve homing and survival of therapeutic stem cells has not been successful.

Previous studies^9, 10, 11^ suggest that protein kinase C *ɛ* (PKC*ɛ*) is essential for signal transduction for ischemic cardioprotection, but whether it has an effect on stem cell retention and survival and what mechanism underlies this effect is uncertain. We know that SDF-1 increased significantly in mesenchymal stem cells (MSCs) after treatment with PKC activator and decreased after treatment with a PKC*ɛ* inhibitor in preliminary experiments, and our latest work indicates that activating PKC*ɛ* improves migration and paracrine function of MSCs *in vitro.*^12^ Thus, we suggest that PKC*ɛ* overexpression in transplanted bone marrow MSCs (BMMSCs) would improve retention and survival of MSC's and improve cardiac function and remodeling in a rat AMI model.

## Results

### Phenotype characterization of BMMSCs

MSCs obtained from rat femurs were amplified and passaged, and MSC vitality was measured using with Trypan blue (91.8%±3.3%). Flow cytometry confirmed CD29 and CD44 expression levels were 98.04% and 98.73%, respectively, and CD34- and CD45-positive cells were 5.50% and 5.35%, respectively, in cultured BMMSCs. Thus, cultured cells were MSCs and not hematopoietic cells.

**Figure 1:**
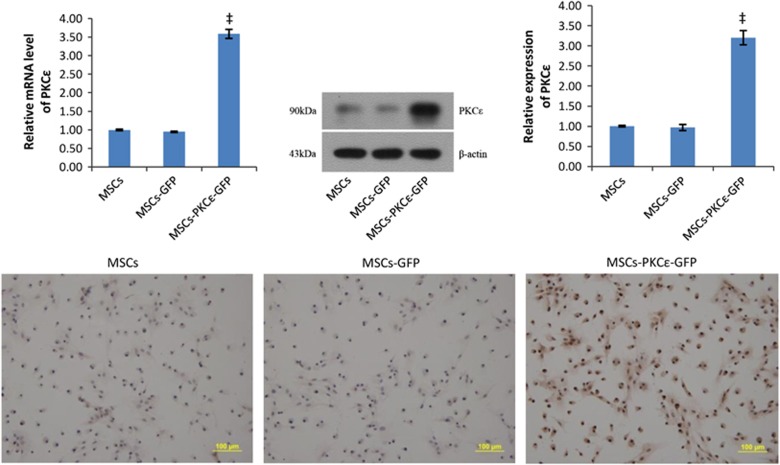
Overexpression of PKC*ɛ* in MSCs. Western blot quantification of PKC*ɛ* expression in different MSCs. Bar graphs show relative mRNA and relative protein expression of PKC*ɛ* in different MSCs. Typical immunocytochemistry images (200 ×) in different MSCs are shown and indicate that PKC*ɛ* (brown) was overexpressed significantly in the MSCs-PKC*ɛ*-GFP group. Cell nuclei are blue. Data are means±S.D. for three different experiments. ^‡^*P*< 0.001 *versus* MSCs-GFP group

Lentiviral transfection efficiency to MSCs was 89.2%±2.3%. Flow cytometry confirmed CD29 and CD44 expression of 97.36% and 97.52%, respectively, and CD34- and CD45-positive cells were 5.32% and 5.44%, respectively, in transfected BMMSCs (Supplementary Figure S1), and this was not significantly different from untransfected MSCs. Analysis by real-time polymerase chain reaction (PCR), western blot and immunocytochemistry indicated that expression of PKC*ɛ* in MSCs-PKC*ɛ*-green fluorescent protein (GFP) increased significantly compared with MSCs-GFP (Figure 1), verifying that PKC*ɛ*-overexpressed MSCs were successfully constructed.

### Animal survival

Rats died in all treatment groups, giving numbers as follows: one for sham; four for AMI; two for MSCs; two for MSCs-GFP, one for MSCs-PKC*ɛ*, two for AMD3100 and three for the LY294002 group. Six animals were killed for analysis early in each group (1 day after transplantation), and at later phases the remaining rats in each group, sham (nine), AMI (six), MSCs (eight), MSCs-GFP (eight), MSCs-PKC*ɛ* (nine), AMD3100 (eight), and LY294002 (seven) were killed for analysis (4 weeks after transplantation).

### Retention of transplanted MSCs

**Figure 2:**
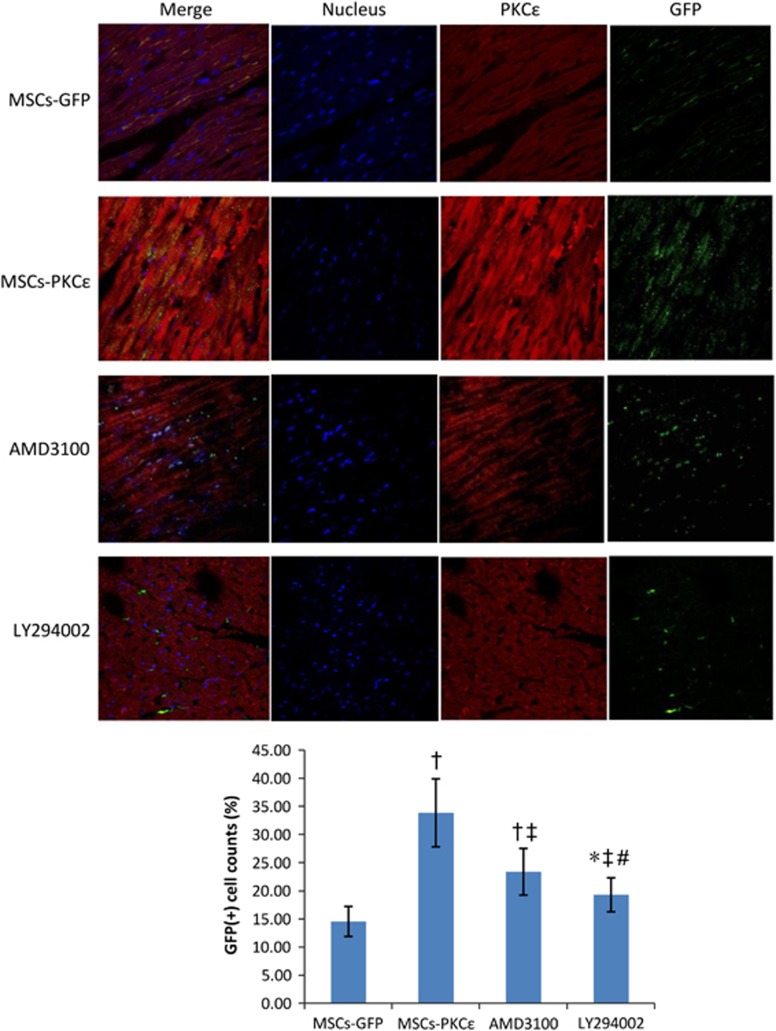
Retention and distribution of transplanted MSCs in each group 1 day after transplantation. Immunofluorescent images (600 ×) from second to fourth arrays indicate cell nuclei (blue), PKC*ɛ* (red) and GFP (green), respectively, and first array images (left side) are merged from second to fourth arrays. Bar graph shows GFP (+) cell count percents [(GFP (+) cell counts/total cell counts) × 100%], indicating that expression of PKC*ɛ* (red) and retention of MSCs (green) increased significantly in the MSCs-PKC*ɛ* group compared with controls, and increases were attenuated after treatment with AMD3100 or LY294002 (*n*=6). Data are means±S.D. for five different experiments (*n*=6). MSCs-GFP: AMI+MSCs-GFP group; MSCs-PKC*ɛ*: AMI+MSCs-PKC*ɛ*-GFP group; AMD3100: AMI+MSCs-PKC*ɛ*-GFP+AMD3100 group; LY294002: AMI+MSCs-PKC*ɛ*-GFP+LY294002 group; *P<0.05 *versus* MSCs-GFP group; ^†^*P*<0.01 *versus* MSCs-GFP group; ^‡^*P*<0.05 *versus* MSCs-PKC*ɛ* group; ^#^*P*<0.05 *versus* AMD3100 group

Immunofluorescent analysis indicated that transplanted MSCs in PKC*ɛ*-overexpressed groups increased 132.44% compared with those in the MSCs-GFP group 1 day after transplantation (*P*<0.05), and this increase was attenuated by 54.22% and 75.4% after treatment with AMD3100 and LY294002, respectively, but transplanted MSCs in AMD3100 and LY294002 groups increased by 60.63% and 32.59% compared with the MSCs-GFP group (*P*<0.05), indicating that activation of PKC*ɛ* enhanced MSC retention and inhibiting the SDF-1/CXCR4 axis or the phosphatidylinositol 3 kinase (PI3K)/protein kinase B (AKT) pathway partly attenuated the effects of PKC*ɛ* (Figure 2).

### Changes in the expression levels of the principal signal protein 1 day after transplantation

**Figure 3:**
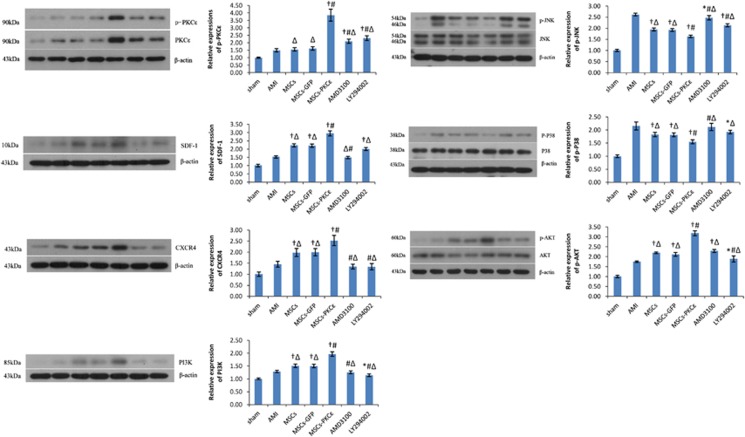
Expression of principal signal proteins in the PKC*ɛ* signaling, SDF-1/CXCR4 axis and PI3K/AKT pathway in each group 1 day after transplantation. Western blots quantification of PKC*ɛ*/p-PKC*ɛ*, SDF-1, CXCR4, PI3K, JNK/pJNK, P38MAPK/p-P38 and AKT/p-AKT. Bar graphs show relative protein expression compared with *β*-actin after densitometric scanning. Data are means±S.D. for three different experiments (*n*=6). Sham: sham group; AMI: AMI group; MSCs: AMI+MSCs group; MSCs-GFP: AMI+MSCs-GFP group; MSCs-PKC*ɛ*: AMI+MSCs-PKC*ɛ*-GFP group; AMD3100: AMI+MSCs-PKC*ɛ*-GFP+AMD3100 group; LY294002: AMI+MSCs-PKC*ɛ*-GFP+LY294002 group; *P<0.05 *versus* AMI group; ^†^*P*<0.01 versus AMI group; ^#^*P*<0.01 *versus* MSCs-GFP group; ^Δ^*P*<0.05 *versus* MSCs-PKC*ɛ* group

Messenger RNA (mRNA) for PKC*ɛ*, SDF-1, CXCR4, PI3K and AKT in PKC*ɛ*-overexpressed animals increased significantly compared with controls (*P*<0.05), and this was partly attenuated by AMD3100 or LY294002 (Supplementary Figure S2). Similarly, protein expression for p-PKC*ɛ*, SDF-1, CXCR4, PI3K, phosphorylated AKT (p-AKT) in PKC*ɛ*-overexpressed animals increased by 137.65%, 34.09%, 26.50%, 30.67% and 50.71% (*P*<0.05 *versus* MSCs-GFP) and these p-PKC*ɛ* increases decreased by 78.48% and 69.06% after AMD3100 or LY294002 treatment, respectively, but p-PKC*ɛ* expression still increased by 29.63% and 42.59% compared with the MSCs-GFP group (*P*<0.05; Figure 3). Thus, activation of PKC*ɛ* signaling activates the SDF-1/CXCR4 axis and the PI3K/AKT pathway, and inhibiting the SDF-1/CXCR4 axis or the PI3K/AKT pathway can partially reduce PKC*ɛ* signaling.

### Alterations in cardiac phenotypes

In echocardiographic studies of the AMI group at 4 weeks, the ejection fraction (EF), percent fractional shortening (FS) and interventricular septum thickness in end-diastole (IVSd) decreased by 34.52%, 39.18% and 50.00%, whereas left ventricle end-diastolic diameter (LVEDD), left ventricle end-systolic diameter (LVESD) and left ventricular mass (LVmass) increased by 62.71%, 51.11% and 37.69% (Table 1) compared with sham (*P*<0.05). In the MSCs-GFP group, EF increased by 20.1% compared with the AMI group, and further increased by 15.8% in the MSCs-PKC*ɛ* group, exceeding that of the MSCs-GFP group (*P*<0.05). LVEDD, LVESD and LVmass decreased by 17.86%, 13.56% and 10.76%, and IVSd increased by 28.57% in the PKC*ɛ*-overexpressed group (*P*<0.05 *versus* MSCs-GFP). Thus, cardiac function and phenotype is improved in the MSCs-PKC*ɛ* group. The beneficial effects of PKC*ɛ* were attenuated by AMD3100 and LY294002 treatment, and improvement of EF in the MSCs-PKC*ɛ* group over that in the MSCs-GFP group decreased by 72.49% and 83.25% in AMD3100 and LY294002 groups, respectively.

Histological assessment suggests that rats in the AMI group had cardiomyocytes that were more disordered, with larger cross-sectional diameters and greater fibrosis with larger infarcts compared shams. MSC transplantation improved these pathological changes, whereas PKC*ɛ*-overexpressed MSC transplantation therapy diminished cardiomyocyte diameter, collagen area, and infarct size by 25.10%, 43.93% and 28.65%, respectively, (*P*<0.05) and produced less fibrosis and more orderly cardiomyocyte arrangement compared with the MSCs-GFP group. AMD3100 and LY294002 treatment partially impaired but did not abolish the beneficial histological effects of PKC*ɛ* (Supplementary Figures S3–S5). Cardiac phenotype changes suggested that overexpression of PKC*ɛ* enhanced the therapeutic effects of MSCs and improved cardiac function and remodeling after AMI. Inhibiting SDF-1/CXCR4 or PI3K/AKT signaling partly impaired the effects of PKC*ɛ*.

### Increased survival of transplanted MSCs

**Figure 4:**
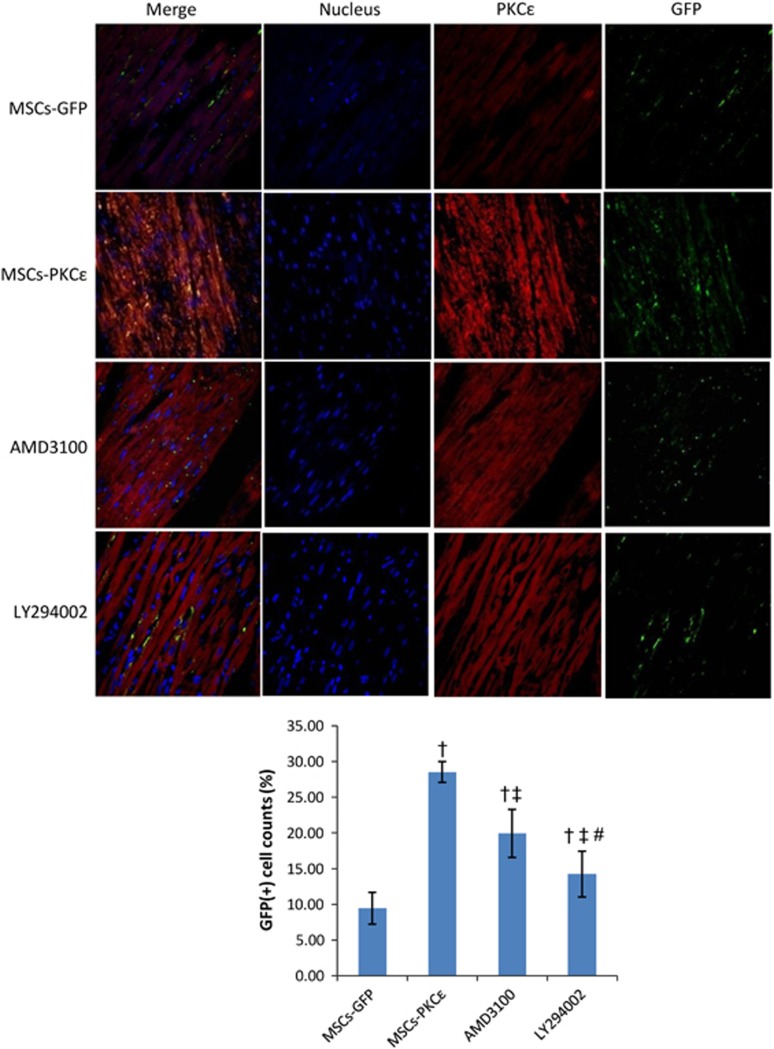
Distribution of transplanted MSCs in each group 4 weeks after transplantation. Immunofluorescent images (600 ×) from second to fourth arrays indicate cell nuclei (blue), PKC*ɛ* (red) and GFP (green), and images of the first array (left side) are merged from second to fourth arrays. Bar graph shows GFP (+) cell count percents [(GFP (+) cell counts/total cell counts) × 100%], indicating that expression of PKC*ɛ* (red) and survival of MSCs (green) increased significantly in MSCs-PKC*ɛ* animals compared with controls and that increases were attenuated after treatment with AMD3100 or LY294002 (*n*=6). Data are means±S.D. for five different experiments (*n*=6). MSCs-GFP: AMI+MSCs-GFP group; MSCs-PKC*ɛ*: AMI+MSCs-PKC*ɛ*-GFP group; AMD3100: AMI+MSCs-PKC*ɛ*-GFP+AMD3100 group; LY294002: AMI+MSCs-PKC*ɛ*-GFP+LY294002 group; ^†^*P*<0.01 *versus* MSCs-GFP group; ^‡^*P*<0.05 *versus* MSCs-PKC*ɛ* group; ^#^*P*<0.05 *versus* AMD3100 group

**Figure 5:**
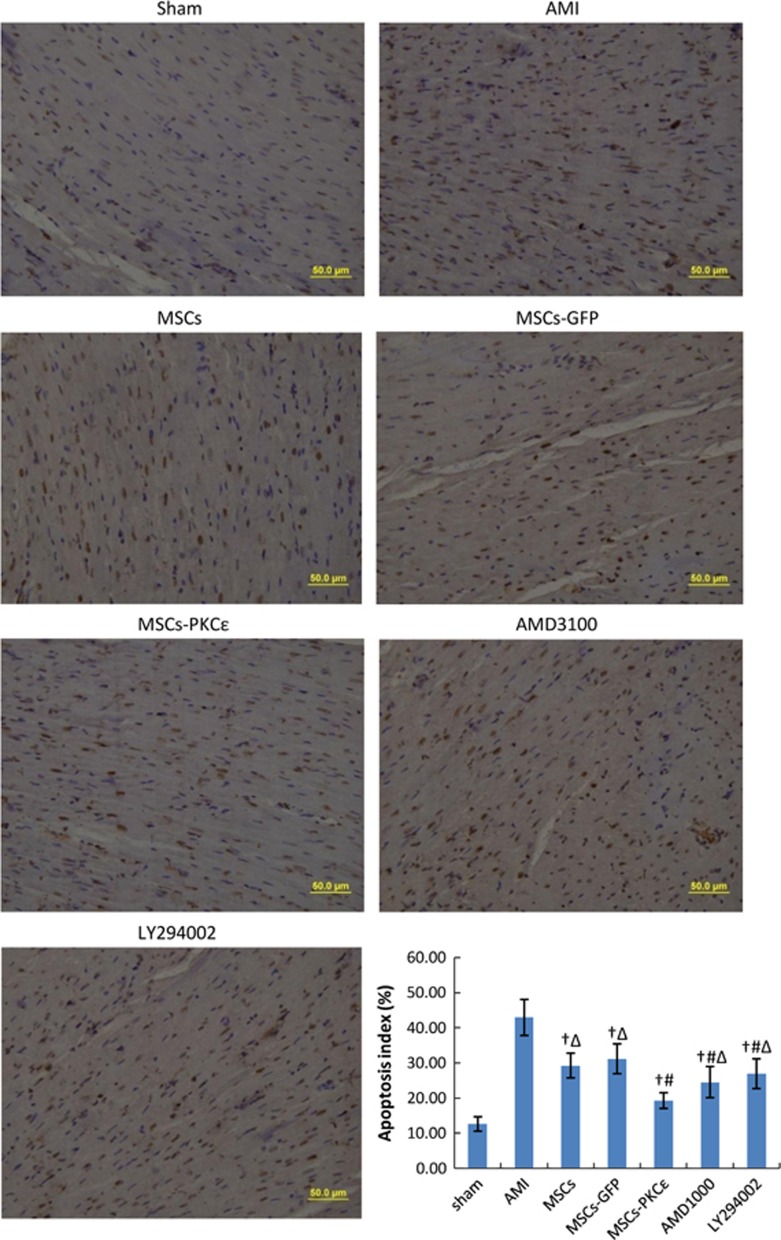
Apoptosis for each group 4 weeks after transplantation (400 ×). Viable cell nuclei appear blue and apoptotic cell nuclei are brown. Bar graph shows apoptosis for each group: index=(apoptotic cell counts/total cell counts) × 100%. Data are means±S.D. for five different experiments (*n*=6). Data show that apoptosis decreased significantly in the MSCs-PKC*ɛ* group compared with other groups, and treatment with AMD3100 or LY294002 impaired the effects of PKC*ɛ*. Sham: sham group; AMI: AMI group; MSCs: AMI+MSCs group; MSCs-GFP: AMI+MSCs-GFP group; MSCs-PKC*ɛ*: AMI+MSCs-PKC*ɛ*-GFP group; AMD3100: AMI+MSCs-PKC*ɛ*-GFP+AMD3100 group; LY294002: AMI+MSCs-PKC*ɛ*-GFP+LY294002 group; ^†^*P*<0.01 *versus* AMI group; ^#^*P*<0.01 *versus* MSCs-GFP group; ^Δ^*P*<0.05 *versus* MSCs-PKC*ɛ* group

Transplanted MSCs in the infarct border zone in the PKC*ɛ*-overexpressed group increased by 2.02-fold 4 weeks after transplantation compared with normal PKC*ɛ* MSCs. PKC*ɛ* effects were impaired by 45.03% and 74.87% after treatment with AMD3100 or LY294002 (*P*<0.05; Figure 4). Apoptosis increased significantly in the AMI group, and activation of PKC*ɛ* in the MSCs-PKC*ɛ* group ameliorated apoptosis by 38.25% compared with the MSCs-GFP group, but this improvement decreased by 44.20% and 64.34% after treatment with AMD3100 or LY294002 (*P*<0.05; Figure 5).

### Alterations in VEGF, bFGF and TGF-*β* expression

**Figure 6:**
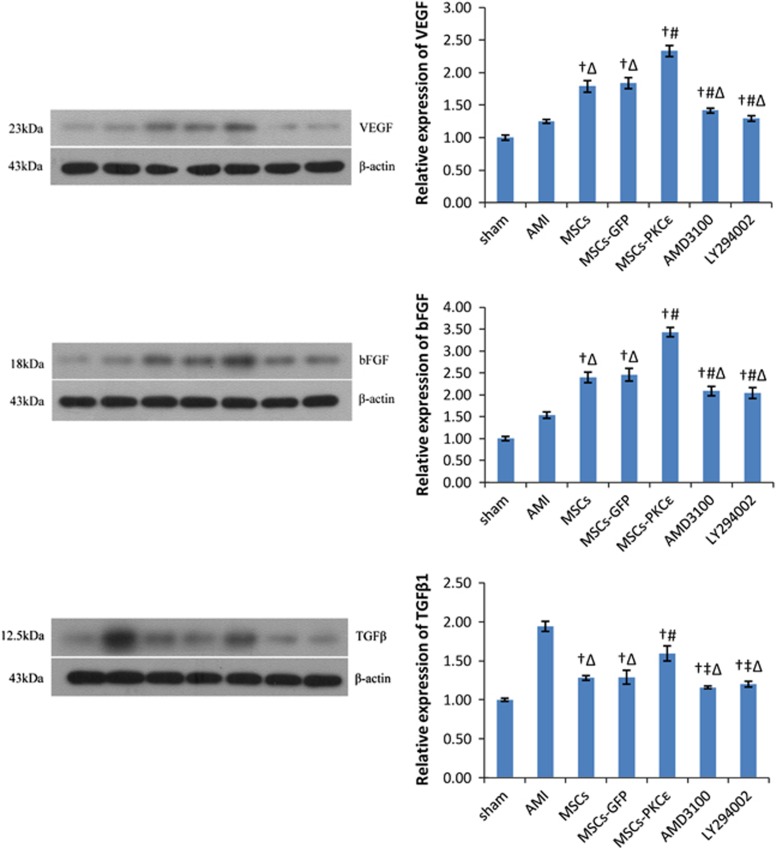
VEGF, bFGF and TGF*β*1 protein expression in each group 4 weeks after transplantation. Western blots quantification of VEGF, bFGF and TGF*β*1 expression. Bar graphs show expression of proteins compared with *β*-actin after densitometric scanning. Data are means±S.D. for three different experiments (*n*=6). Sham: sham group; AMI: AMI group; MSCs: AMI+MSCs group; MSCs-GFP: AMI+MSCs-GFP group; MSCs-PKC*ɛ*: AMI+MSCs-PKC*ɛ*-GFP group; AMD3100: AMI+MSCs-PKC*ɛ*-GFP+AMD3100 group; LY294002: AMI+MSCs-PKC*ɛ*-GFP+LY294002 group; ^†^*P*<0.01 *versus* AMI group; ^‡^*P*<0.05 *versus* MSCs-GFP group; ^#^*P*<0.01 *versus* MSCs-GFP group; ^Δ^*P*<0.05 *versus* MSCs-PKC*ɛ* group

Overexpression of PKC*ɛ* enhanced mRNA and protein expression of vascular endothelial growth factor (VEGF) and basic fibroblast growth factor (bFGF) in the transplantation zone (Supplementary Figure S6 and Figure 6), even after corrections for cell numbers, indicating that PKC*ɛ* increased paracrine factors related to growth and angiogenesis. However, expression of transforming growth factor *β*1 (TGF*β*1), which is related to ventricular remodeling after AMI, was greatest in the AMI group, and overexpression of PKC*ɛ* decreased expression of TGF*β*1 by 18.04% (*P*<0.05 *versus* AMI; Figure 6), which may be associated with improved cardiac remodeling in the MSCs-PKC*ɛ* group.

### Changes in cTnI, vWF and SMA expression

**Figure 7:**
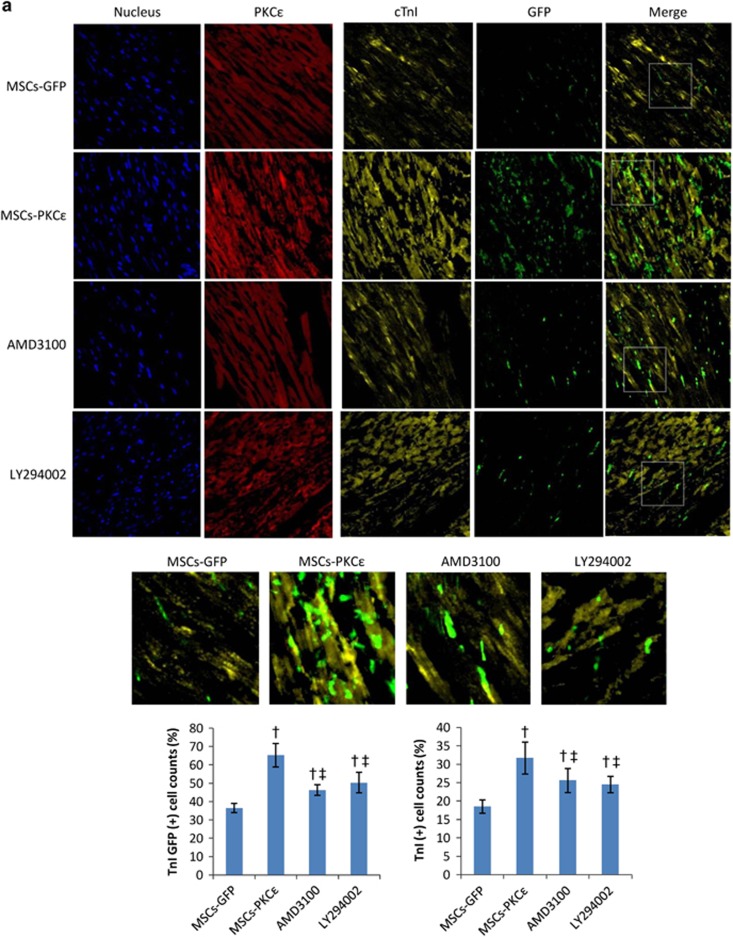

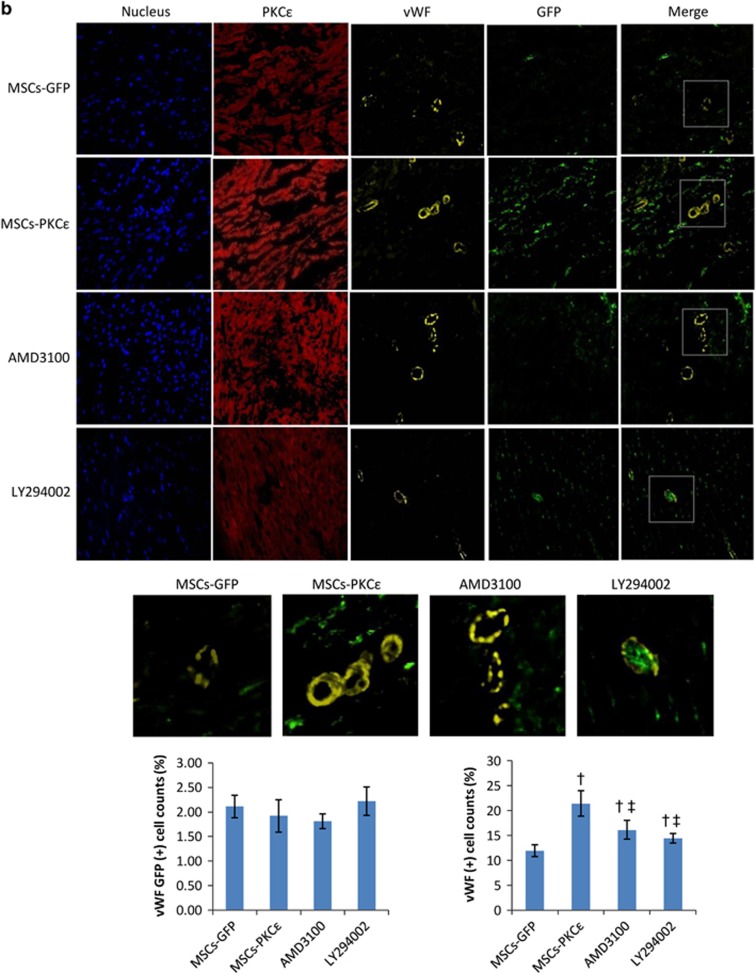

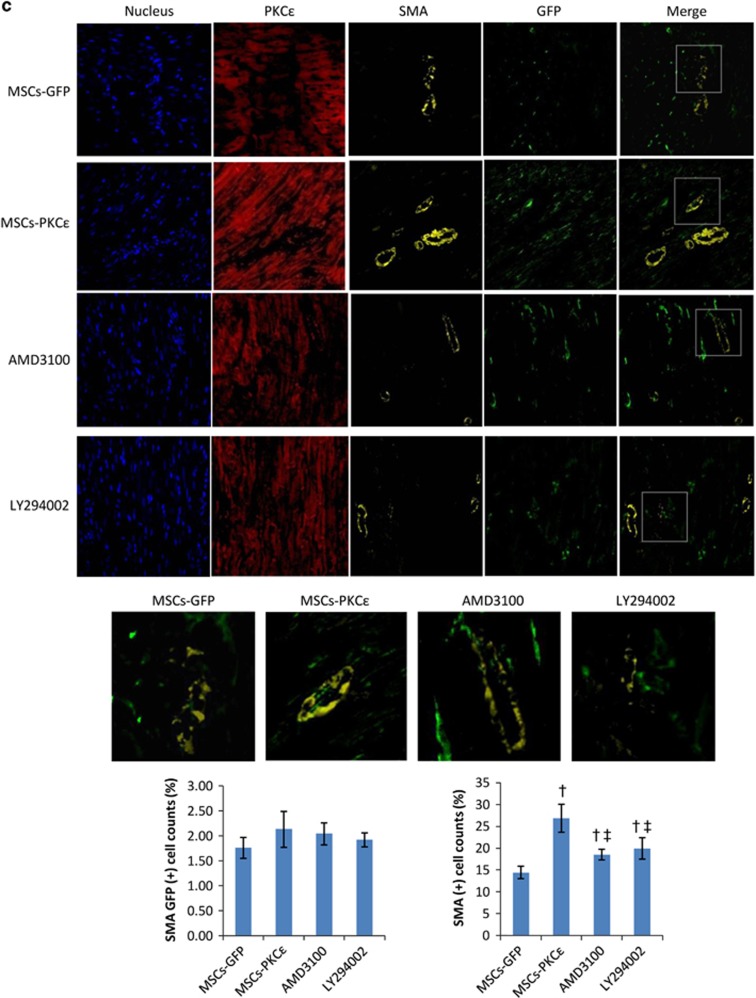
Expression of cTnI (**a**), vWF (**b**) and SMA (**c**) in each group 4 weeks after transplantation. Immunofluorescent images (600 ×) from first to fourth arrays indicate cell nuclei (blue), PKC*ɛ* (red), interest protein (cTnI/vWF/SMA, yellow) and GFP (green), respectively. Images of the first array (right side) are merged from third and fourth arrays and show colocalization of proteins of interest and GFP-positive MSCs. Part of the merged image was amplified to show colocalization. Bar graphs show proteins of interest and GFP (+) cell count percents [(interest protein and GFP double (+) cell counts/GFP (+) cell counts) × 100%] and protein of interest (+) cell count percents [(interest protein (+) cell counts/total cell counts) × 100%], respectively. Data are means±S.D. for five different experiments (*n*=6). Data show that cTnI/vWF/SMA (yellow) (+) cells increased significantly in the MSCs-PKC*ɛ* group, and cTnI and GFP colocalized cells increased significantly in the MSCs-PKC*ɛ* group, but there was little colocalization of vWF/SMA and GFP in each group, suggesting that PKC*ɛ* could contribute to differentiation of MSCs into myocardial cells and increase myocyte, vascular endothelial cell, and vascular smooth muscle cell survival but PKC*ɛ* had no effect on differentiation of MSCs into vascular cells and increased survival of vascular cells in the MSCs-PKC*ɛ* group is likely due to enhanced paracrine effects and not increasing differentiation of MSCs. Increased expression of cTnI/vWF/SMA was attenuated after treatment with AMD3100 or LY294002 (*n*=6). MSCs-GFP: AMI+MSCs-GFP group; MSCs-PKC*ɛ*: AMI+MSCs-PKC*ɛ*-GFP group; AMD3100: AMI+MSCs-PKC*ɛ*-GFP+AMD3100 group; LY294002: AMI+MSCs-PKC*ɛ*-GFP+LY294002 group; ^†^*P*<0.01 *versus* MSCs-GFP group; ^‡^*P*<0.05 *versus* MSCs-PKC*ɛ* group

Overexpression of PKC*ɛ* increased the percentage of cardiac troponin I (cTnI) GFP (+) (colocalized cells of cTnI and GFP) and cTnI (+) cells by 78.90% and 71.35%, respectively, and the distribution of cTnI was related to expression of PKC*ɛ*, indicating that PKC*ɛ* participates in and contributes to differentiation of MSCs into cardiomyocytes and their survival. In addition, overexpression of PKC*ɛ* increased the percentage of von Willebrand factor (vWF) (+) and smooth muscle actin (SMA) (+) cells by 79.83% and 86.11%, but there was little colocalization of vWF/SMA and GFP in each group, suggesting that PKC*ɛ* can increase the survival of vascular endothelial and vascular smooth muscle cells, but PKC*ɛ* had no effect on MSC differentiation into vascular cells. Increased vascular cell survival in the MSCs-PKC*ɛ* group was likely due to enhanced paracrine effects of MSCs and not increased differentiation of MSCs into vascular cells. AMD3100 or LY294002 treatment impaired the effects of PKC*ɛ*, and the increases in the percentage of vWF (+) cells decreased by 55.79% and 73.68% and the increases in the percentage of SMA (+) cells decreased by 66.94% and 55.65% in AMD3100 or LY294002 groups, respectively, (*P*<0.05 *versus* MSCs-PKC*ɛ*; Figure 7).

### Neovessel changes

**Figure 8:**
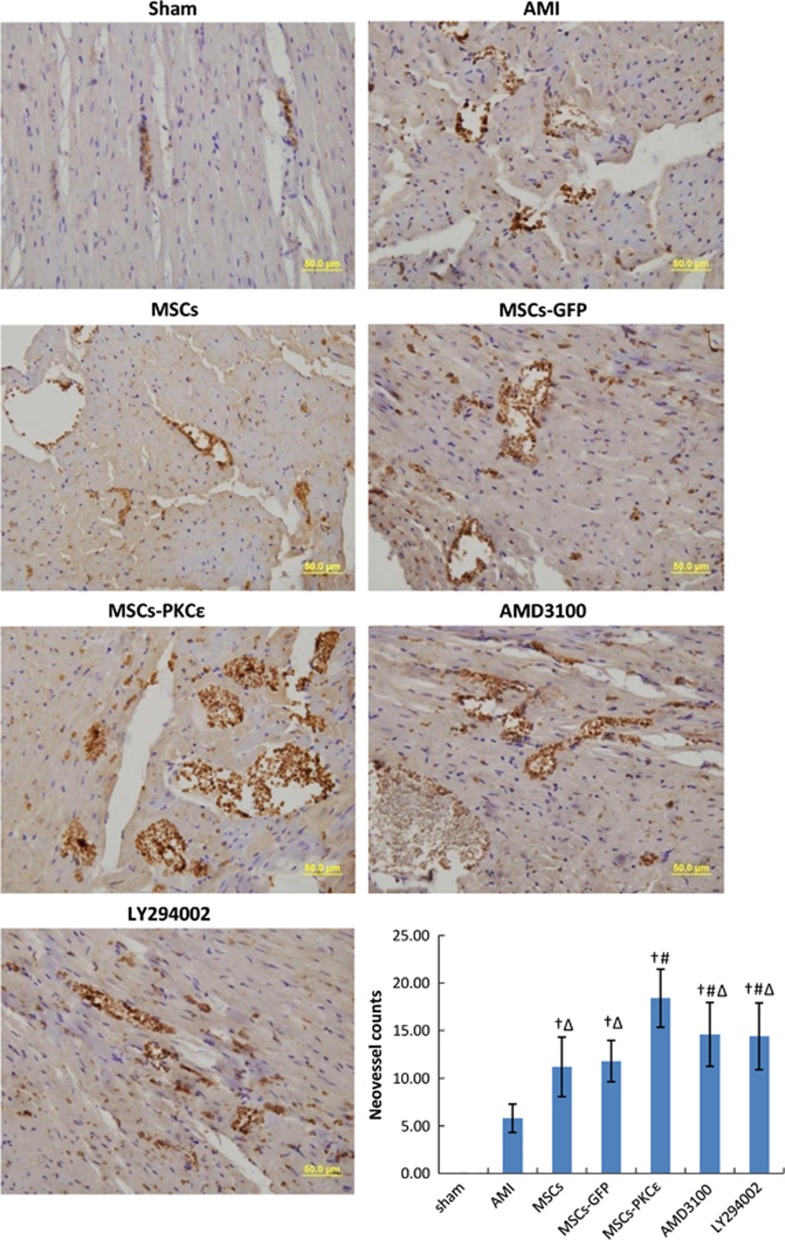
Expression of factor VIII in each group at 4 weeks after transplantation (400 ×). Cell nuclei appear blue and factor VIII stains brown. Bar graph shows neovessel counts per group as assessed microscopically (400 ×), and data are means±S.D. for five different experiments (*n*=6). Data show that expression of factor VIII and neovessel counts increased significantly in the MSCs-PKC*ɛ* group compared with controls, suggesting that PKC*ɛ* contributed to expression of factor VIII and formation of neovessels. These effects were partly inhibited after treatment with AMD3100 or LY294002. Sham: sham group; AMI: AMI group; MSCs: AMI+MSCs group; MSCs-GFP: AMI+MSCs-GFP group; MSCs-PKC*ɛ*: AMI+MSCs-PKC*ɛ*-GFP group; AMD3100: AMI+MSCs-PKC*ɛ*-GFP+AMD3100 group; LY294002: AMI+MSCs-PKC*ɛ*-GFP+LY294002 group; ^†^*P*<0.01 *versus* AMI group; ^#^*P*<0.01 *versus* MSCs-GFP group; ^Δ^*P*<0.05 *versus* MSCs-PKC*ɛ* group

Factor VIII expression increased significantly and neovessels increased by 55.93% in PKC*ɛ*-overexpressed animals compared with the MSCs-GFP group. Treatment with a CXCR4 antagonist or a PI3K inhibitor attenuated the effects of PKC*ɛ* by 57.58% and 60.61%, respectively, (*P*<0.05; Figure 8).

## Discussion

Here we report that PKC*ɛ* effects retention and survival of MSCs and improves cardiac function and remodeling in rat AMI models. So, we studied changes in principal proteins in the PKC*ɛ* signaling, the SDF-1/CXCR4 axis and the PI3K/AKT pathway under different conditions involving activation of PKC*ɛ*, CXCR4 antagonism and PI3K inhibition. We observed that overexpression of PKC*ɛ* significantly increased retention, survival and differentiation of transplanted MSCs, improved cardiac function and remodeling, decreased apoptosis and infarct size, and increased production of important paracrine factors and neovessel formation. These factors presumably enhanced the effects of MSC transplantation and PKC*ɛ* effects were partially independent of the SDF-1/CXCR4 axis and PI3K/AKT pathway.

The SDF-1/CXCR4 axis is important for homing, survival and differentiation of stem cells.^13, 14, 15, 16, 17^ Decreasing degradation of SDF-1,^13^ pretreatment with SDF-1^14, 15^ or increasing expression of SDF-1 and CXCR4 by gene modification^16, 18^ all increase homing and enhance stem cell therapeutic effects. Hypoxic pretreatment also increases CXCR4 and recruits stem cells to zones of ischemic damage.^17^ In addition, matching the time of expression of SDF-1 and CXCR4 improves stem cell effects.^17^ Previous work^19^ indicates that SDF-1/CXCR4 may mediate migration of BMMSCs via activation of PI3K/AKT, a known cytoprotective pathway. Another *in vitro* study indicated that supplemented/conditioned media (without stem cells) could attenuate myocardial reperfusion injury and cardioprotection may be mediated by activating the PI3K pathway via paracrine factors.^20^ Thus, these data suggest that the PI3K/AKT pathway may be critical for migration and paracrine effects of BMMSCs.

PKC*ɛ* signaling is important for ischemic cardioprotection,^9^ and studies indicate that activation of PKC*ɛ* can attenuate reperfusion injury, increase resistance to ischemia, diminish infarct size and improve cardiac function by activating serial downstream proteins, mitogen-activated protein kinase (MAPK), extracellular regulated protein kinase, lymphocyte cell kinase and Src family kinase.^11, 21, 22^ Inhibiting PKC*ɛ* abolished these protective effects,^10^ so activating PKC*ɛ* appears to be critical for ischemic cardioprotection. The roles of PKC*ɛ* in hypertrophy and heart failure have been verified and its signaling complexes have been identified by us previously.^23^ However, the effect of PKC*ɛ* on retention and survival of MSCs and assessment of potential molecular mechanisms for this has not been established. Our latest data indicate that activation of PKC*ɛ* can improve migration and increase production of important paracrine factors (including prosurvival factors, critical growth factors, modulating factors of inflammation, etc.) of MSCs *in vitro*, partly independent of the SDF-1/CXCR4 axis and the PI3K/AKT pathway.^12^ We verified that activation of PKC*ɛ* also can enhance SDF-1/CXCR4 axis and PI3K/AKT pathway activity in MSCs *in vivo*.

Although activation of PKC induced upregulation of c-Jun N-terminal kinase (JNK)/phosphorylated JNK (pJNK)/P38 in MSCs *in vitro,*^12^ downregulation of pJNK/P38 expression was observed in the PKC*ɛ*-overexpressed group *in vivo*, which was probably associated with negative feedback regulation of JNK/P38 signaling induced by persistent activation of PKC*ɛ in vivo*. Previous studies show that overexpression of JNK/P38MAPK can contribute to apoptosis,^24, 25, 26, 27^ hypertrophy and remodeling,^28, 29, 30, 31^ and attenuate contractility.^32, 33^ This could contribute to development of heart failure and this idea is in agreement with our current results.

Both western blot and cardiac phenotype suggested that animals in the PI3K inhibitor group had worse outcomes compared with the CXCR4 antagonist group, likely because the effects of the PI3K/AKT pathway (including inhibiting apoptosis, contributing to cell migration, proliferation and survival) were attenuated. Alternatively, PKC*ɛ* signaling was partly inhibited by cross-talking with the PI3K/AKT pathway and cardioprotective effects of PKC*ɛ* were also diminished.

Persistent problems with stem cell therapy are poor homing and survival, and even with advances in tissue-engineering,^13, 14, 15, 16, 17, 34, 35^ substantial breakthroughs for homing and survival are lacking. Our previous data indicate that activation of PKC*ɛ* can enhance motility and secretion of paracrine factors involved in migration, proliferation and differentiation of MSCs *in vitro.*^12^ Here, we revealed for the first time that activating PKC*ɛ* enhances retention, paracrine function related to growth and angiogenesis, and survival of MSCs *in vivo* and that these are positive effects in a rat AMI model.

Previous work indicates that MSC transplantation can improve cardiac function after AMI and we show that overexpression of PKC*ɛ* further improved cardiac function and ventricular remodeling in association with decreased apoptosis, increased retention, survival and differentiation of MSCs and formation of neovessels. Increases in LVmass and LVEDD in the PKC*ɛ*-overexpressed group were less than those in other groups, suggesting that PKC*ɛ* signaling can delay the development of ventricular hypertrophy and remodeling. Previous data indicate that MSC therapy can decrease infarct size, and our work shows that PKC*ɛ* activation can further decrease infarct areas, a finding significant to prevention of subsequent heart failure after AMI.

Exogenous MSCs could activate resident cardiac stem cells (CSCs), enhance proliferation, mobilization, differentiation, survival, angiogenesis and function of CSCs, or even restore CSC niches by secreting paracrine factors including hepatocyte growth factor, SDF-1, VEGF and insulin-like growth factors 1.^5^ As paracrine function was enhanced by PKC*ɛ* activation, the effects of MSCs on CSCs also are conceivably enhanced when PKC*ɛ*-overexpressed MSC therapy is used for AMI. In addition, overexpression of TGF*β*1 can induce interstitial fibrosis, and this relates to ventricular remodeling after AMI via activation of Smad3.^36, 37^ However, moderate collagen deposition and fibrosis is necessary to compensate for and replace lost myocardial cells and necrotic cardiac tissue after AMI, and too little collagen deposition after AMI can cause additional deterioration of cardiac function, especially reduced myocardial collagen cross-linking.^38^ In addition, TGF-*β* can mediate myogenic differentiation of BMMSC and induce therapeutic angiogenesis, contributing to functional cardiac regeneration.^39^ With PKC*ɛ*-overexpressed MSC transplantation therapy for AMI, beneficial effects of PKC*ɛ* on remodeling may be partly associated with greater expression of TGF-*β* in the MSCs-PKC*ɛ* group, which may increase cardiomyogenic differentiation of MSCs and angiogenesis, contributing to cardiac regeneration and an improved phenotype.

### Clinical implications

Previous studies prove that activation of PKC*ɛ* can lower ischemic/reperfusion injury and improve cardiac function perhaps by enhancing mitochondrial oxidative phosphorylation,^40^ and by inactivating pro-apoptotic proteins and inducing expression of bcl-2.^41^ Whether PKC*ɛ* has an effect on stem cell therapy and how this occurs if so is uncertain, but our work verified that activating PKC*ɛ* can improve retention and survival of MSCs directly and indirectly. Thus, reinforcing PKC*ɛ* signaling can work in multiple ways to attenuate ischemic injury, including enhancing MSC transplantation effectiveness, which suggests that selectively activating PKC*ɛ* with drugs or gene therapy may significantly improve ventricular remodeling and cardiac function and prognosis after AMI by inducing multiple cardioprotection when MSC transplantation therapy are used for AMI. PKC*ɛ* may be a novel target for AMI therapy. Future studies to understand the effects of PKC*ɛ* on human CSCs and heart function after AMI and how PKC*ɛ* affects the differentiation of MSCs are underway, and these will inform us about the role of and the molecular mechanisms behind PKC*ɛ* in stem cell therapy.

### Conclusion

Activation of PKC*ɛ* improves retention, survival and differentiation of MSCs and enhances their therapeutic effects in AMI. PKC*ɛ* pathway intervention may be a therapeutic target for improving stem cell therapy.

## Materials and Methods

The study was reviewed and approved by the Institutional Ethics Committee on Animal Resources of the Anzhen Hospital and Beijing Institute of Heart, Lung, and Blood Vessel Diseases, and conformed to the guiding principles of the ‘Guide for the Care and Use of Laboratory Animals' (NIH publication no. 83-23, revised 1996).

### Isolation, expansion and passage of rat BMMSCs

BMMSCs were isolated and passaged as described previously.^42^ Briefly, the femurs of Sprague–Dawley rats (male, 100–120 g) were collected after killing animal by cervical dislocation, and BMMSCs were separated with Histopaque-1083 separating medium (Sigma-Aldrich, St. Louis, MO, USA). Cells were centrifuged at 1000 × *g* for 10 min and suspended in Dulbecco's modified Eagle's medium with low glucose (GIBCO, Carlsbad, CA, USA) supplemented with 10% fetal bovine serum (Hyclone, Logan, UT, USA). After cells were vaccinated in a culture flask (2 × 10^5^ cells/cm^2^) and incubated at 37 °C with 5% CO_2_ and saturated humidity, the adherent layer was washed once every 2 days with fresh medium. Cells from passages 4–8 were harvested and used in subsequent biochemical experiments.

### Viability assay and growth of MSCs

MSCs viability was measured with Trypan blue exclusion assay; viable cells were counted. Cell viability (%) was calculated as the percentage of cells lacking dye. MSCs from passage 5 were digested with 0.25% trypsin and added to 96-well plates (2 × 10^4^ cells/ml; 200 *μ*l per well) and cultured for 8 consecutive days. The optical density (OD) and cell counts were measured daily. OD (570 nm) was measured after incubating cells with 20 *μ*l 5 mg/ml methyltetrazolium for 4 h followed by addition of 150 *μ*l DMSO. A culture solution without cells was a blank control. Cell counts were performed using Trypan blue and growth curves were made. Population doubling time (T_D_)=t[log_2_/(logNt–logN_0_)].

### Identification of MSCs surface markers by flow cytometry

After cells were digested with 2.5 g/l trypsin, MSCs were prepared (1 × 10^6^/ml) and incubated for 30 min at 37 °C with monoclonal antibodies against CD29, CD44, CD34 and CD45. Cells were centrifuged and washed three times with PBS and incubated for 30 min with the corresponding FITC-labeled secondary antibody (see Supplementary Tables S2 and S3 for antibody data). Homologous IgG and PBS were negative controls. Expression levels of MSC surface markers were analyzed by flow cytometry.

### Construction of PKC*ɛ*-overexpressed MSCs by gene transfer

PKC*ɛ*-overexpressed lentiviruses were established as described previously.^43^ Briefly, cDNA fragments of PKC*ɛ* was obtained by PCR and cloned into the lentiviral vector (pLenti7.3/V5-TOPO) carrying an enhanced GFP (EGFP) reporter gene. The successful construction of PKC*ɛ*-overexpressed vector was verified by sequencing. Then packaged and transferring vectors were co-transfected into 293T cells. Thus, lentiviral solutions – lenti-PKC*ɛ*-EGFP and lenti-EGFP solutions – were obtained. Virus titer was measured and MSCs from passage 4 were plated and cultured until 80% confluent and were infected with lenti-PKC*ɛ*-EGFP or lenti-EGFP solution. Spare virus particles were removed by changing fresh medium. After 24 h, uninfected MSCs were removed by adding 2 *μ*g/ml puromycin (puromycin resistance gene had been inserted into lentiviral vectors) and incubating for another 24 h. Then, two MSCs, MSCs-GFP and MSCs-PKC*ɛ*-GFP, were obtained. The surface markers of MSCs were identified by flow cytometry. After another two passages, transfected MSCs (passage 7) were used for transplantation. Optimal multiplicity of infection (virus particle counts/cell counts) and transfection efficiency were measured. Transfection efficiency=(cell counts expressing GFP/total cell counts) × 100%. Expression of PKC*ɛ* in MSCs was verified by real-time PCR, western blot and immunocytochemistry.

### Establishment of a rat AMI model

A rat AMI model was established by ligating the left anterior descending branch (LAD) of the coronary artery as described previously.^44^ All rats were anesthetized with 1% pentobarbital (40 mg/kg, i.p.) before surgery. Tracheal cannula and ventilators were connected for respiratory support, and the skin was incised along the left sternal border and the third and fourth ribs were clipped, as hearts were exposed. Then, LADs were ligated. To obtain a standardized moderate infarct size in each group, we ligated LADs at 1.5 mm below the level of the inferior margin of the left auricle to achieve infarction sizes of 30–40% with acceptable death rate. The myocardium in the anterior wall and the apex of the heart below the ligation point became white and had significant hypomotility. ST segment elevation exceeded 1 mv in lead I and lead AVL after successful ligation of LAD. Electrocardiogram monitoring and fluid supplements were administrated during surgery, and heat preservation and prophylactic anti-infection therapy with penicillin were given for 3 days after surgery. Identical procedures were performed except LAD ligation in the sham group.

### Experimental protocol *in vivo*

Rats (8 weeks of age) were divided into seven treatment groups (*n*=16): sham group (sham); AMI group (AMI); AMI+MSCs group (MSCs); AMI+MSCs-GFP group (MSCs-GFP); AMI+MSCs-PKC*ɛ*-GFP group (MSCs-PKC*ɛ*); AMI+MSCs-PKC*ɛ*-GFP+AMD3100 (CXCR4 antagonist) group (AMD3100); and the AMI+MSCs-PKC*ɛ*-GFP+LY294002 (PI3K inhibitor) group (LY294002). One hour after LAD ligation, treatment solution was injected into five different sites in the infarct border zone (1 × 10^6^ MSCs, 20 *μ*l for each injection site) located at the juncture between white and hypokinetic myocardium and normal myocardium. Suture marks were made at the central points of transplanted zones. Culture media without MSC's were control injections (sham and AMI group). The treatment solutions for the MSCs, MSCs-GFP and MSCs-PKC*ɛ* groups contained MSCs, MSCs-GFP and MSCs-PKC*ɛ*-GFP, respectively. MSCs in the AMD3100 and LY294002 groups were pretreated with AMD3100 and LY294002, respectively, for 24 h before being administered.

After transplantation (24 h), rats were killed using 3% pentobarbital sodium (100 mg/kg, i.p.), and hearts were harvested and cardiac tissues in the infarct border zones were collected to perform subsequent biochemical analysis. Retention of transplanted MSCs and association with PKC*ɛ* distribution were measured with immunofluorescent staining and we measured mRNA, and protein expression of signal proteins in PKC*ɛ*, SDF-1/CXCR4 and PI3K/AKT pathways using real-time PCR and western blot.^45^

Four weeks after transplantation, echocardiographic measurements were performed on all rats and subsets of animals were killed as mentioned above. Distributions of transplanted MSCs were analyzed by immunofluorescence to determine the effects of PKC*ɛ* on MSC survival. Apoptotic analysis was performed with TdT-mediated dUTP nick end labeling (TUNEL) assay and mRNA and protein expression of VEGF, bFGF and TGF*β* were quantified with real-time PCR and western blot, respectively. cTnI, vWF and SMA are specifically expressed in myocytes, vascular endothelial cells and vascular smooth muscle cells, respectively, and their expression, distribution and relationship with transplanted MSCs were analyzed by immunofluorescence to ascertain effects of PKC*ɛ* on cardiac differentiation of MSCs. Myocardial tissue sections and vascular endothelial cell marker factor VIII were quantified with immunohistochemistry, and vessels were counted and vessel densities were measured. Histology was also used to assess cardiac tissue.

### Echocardiographic measurements

Echocardiographic measurements were performed on all rats before ligation and at 4 weeks after transplantation, and EF, FS, LVEDD and LVESD, IVSd and LVmass were measured using a HP SONOS 5500 sector scanner (Hewlett Packard CO, Andover, MA, USA) with a 7.5 MHz imaging transducer.

### Real-time reverse transcription-PCR analysis of mRNA of principal proteins

Total RNA was extracted from tissue homogenate in each group with RNA Simple Total RNA Kit (TIANGEN, Peking, China). cDNA was prepared using an iScript cDNA Synthesis Kit (Bio-Rad, Hercules, CA, USA) and real-time PCR was performed on samples using an Exicycler 96 (Bioneer, Daejeon, Korea) according to the manufacturer's protocol. mRNA expression of proteins of interest were measured. Primer sequences used are listed in Supplementary Table S1. Amplification and melting curves were obtained, and *β*-actin was the reporter gene. A simple primer reaction product was a negative control.

### Western blot to quantify principal protein expression

Samples were plated in 24-well dishes (50 000 cells per well) and harvested in 50 μl sample buffer, boiled and sonicated. Protein lysates were separated with 10% sodium dodecyl sulfate polyacrylamide gel electropheresis and gels were transferred polyvinylidene fluoride membranes and blocked with nonfat milk (5% w/v). Then, blots were incubated with primary antibodies overnight at 4 °C, followed by incubation with horseradish peroxidase-conjugated secondary antibody for 45 min at 37 °C. Bands were measured with enhanced chemiluminescence (Applygen, Peking, China) and quantified using laser densitometry with a Typhoon 9400 fluorescent scanner and ImageQuant TL 5.0 software (GE Healthcare, Buckinghamshire, UK) as previously described.^45^ *β*-Actin was an internal reference (antibody information see Supplementary Table S2).

### Immunofluorescence analysis

After fixing in acetone solution at 4 °C for 15 min, cryosections of cardiac tissue were washed three times with PBS and blocked with goat serum for 30 min and then incubated with primary antibodies at 4 °C overnight. After washing three times, cryosections were incubated with fluorescent-labeled secondary antibodies (Supplementary Table S4) in the dark for 90 min, and then counterstained with 4',6-diamidino-2-phenylindole. An anti-fluorescence quench agent was added to the cryosections and sections were observed under a Confocal Laser Scanning Microscope (Olympus, Tokyo, Japan). The distribution of transplanted MSCs and protein expression was assessed with Image-Pro Plus 6.0 software (Media Cybernetics, Rockville, MD, USA), and GFP-positive cells and protein-positive cells were counted.

### Apoptosis assay

TUNEL assay was used with an *In Situ* Cell Death Detection Kit (Roche, Basel, Switzerland) according to the provider instructions (Supplementary Table S5). Tissue samples in each group were embedded in paraffin, and sections were made and incubated 60 min with TUNEL reaction mixture at 37 °C in the dark. Samples were then incubated for 30 min with converter-peroxidase solution at 37 °C followed by coloring with diaminobenzidine (DAB) and counterstaining with hematoxylin. Apoptosis was quantified microscopically and an apoptosis index was calculated.

### Immunohistochemistry

After deparaffination, cardiac tissue sections were incubated in 3% H_2_O_2_ for 15 min and blocked with goat serum for 15 min and then incubated with primary antibody, factor VIII antibody overnight at 4 °C. After washing three times, sections were incubated with biotin-labeled secondary antibodies at 37 °C for 30 min, and sections were incubated with horseradish peroxidase labeled SA streptavidin at 37 °C for 30 min. Then, sections were stained with DAB and counterstained with hematoxylin. Factor VIII expression and vessel counts were quantified microscopically (for antibody information see Supplementary Table S6).

### Histological studies

All rats were killed as described above after echocardiographic measurements 4 weeks after transplantation. Myocardial infarct size was measured by 2,3,5,triphenyl-2H-tetrazolium chloride (TTC) as described previously.^46^ Briefly, after echocardiographic study, the LAD was re-occluded, and patent blue dye was administered intravenously to stain normal regions of the left ventricle (LV), and the heart was rapidly excised. LV tissue was isolated and cut into approximately 10 cross-sectional pieces of equal thickness. Nonstained LV areas at risk (AAR) were separated from the surrounding blue-stained LV normal zone, and both regions were separately incubated for 15 min at 37 °C in 1% TTC in 0.1 M phosphate buffer adjusted to pH 7.4. Tissues were fixed overnight in 10% formaldehyde. AAR and blue-stained LV normal zones were weighed to assess AAR/LV. TTC stains living tissue a deep red color, but necrotic tissue appears white within the AAR slices. Each slice was scanned with a commercial scanner (Canoscan LiDE 60, Canon, Tokyo, Japan), and infarcted and noninfarcted areas were measured using an image analysis program. Myocardial infarct size was expressed as a percentage of the AAR.

In addition, rat heart tissues were fixed in neutral formalin for histology and embedded in paraffin. Then, 5 *μ*m serial cross sections were made and stained with hematoxylin and eosin. Five fields and 20 myocardial cells per field were selected randomly, and average diameters were calculated. Observations were made on coded samples by an independent observer blinded to animal identify. After deparaffination, sections of cardiac tissue were dripped into 1% Picro sirius red saturated trinitrophenol solution for 1 h followed by washing for 5 min. Then sections were counterstained with hematoxylin solution for 5 min followed by washing for 1 min. Sections were put in 1% hydrochloric acid alcohol for 3 s followed by washing for 20 min. Collage expression levels in each group were measured microscopically and myocardial interstitial collagen area percents [(collagen area/total area) × 100%] were derived.

### Statistical analysis

All values are means±S.D. Differences in continuous variables between two groups were analyzed via the Student's *t*-test, and differences among three or more groups were evaluated via one-way ANOVA with Bonferroni correction. Differences in categorical data were assessed using a Chi-square test, or in case of low cell counts (<5), Fisher's exact test was used; *P*<0.05 was considered to be significant.

## Figures and Tables

**Table 1:** Echocardiographic parameters in rats in each group

	**Sham (*****n*****=9)**	**AMI (*****n*****=6)**	**MSCs (*****n*****=8)**	**MSCs-GFP (*****n*****=8)**	**MSCs-PKC*****ɛ*** (***n*****=9)**	**AMD3100 (*****n*****=8)**	**LY294002 (*****n*****=7)**
EF, %	67.24±5.82	44.03±4.36	54.38±5.34^†Δ^	53.00±5.20^*Δ^	61.36±6.17^†#^	55.3±4.54^†Δ^	54.4±4.81^†Δ^
FS, %	33.28±2.42	20.24±2.12	24.35±3.34^*Δ^	23.56±3.20^*Δ^	28.67±3.17^†#^	25.2±2.09^†Δ^	25.13±3.28^†‡^
LVEDD, cm	0.59±0.06	0.96±0.10	0.82±0.09^†Δ^	0.84±0.07^†Δ^	0.69±0.08^†#^	0.83±0.09^†Δ^	0.86±0.08*^Δ^
LVESD, cm	0.45±0.03	0.68±0.08	0.55±0.06^†^	0.59±0.03*^Δ^	0.51±0.09^†#^	0.59±0.07*^Δ^	0.57±0.05^†^
IVSd, cm	0.20±0.02	0.10±0.03	0.15±0.02^†Δ^	0.14±0.02^†Δ^	0.18±0.04^†‡^	0.13±0.03*^Δ^	0.14±0.04^†Δ^
LVmass, g	1.30±0.08	1.79±0.13	1.59±0.12^†Δ^	1.58±0.10^†Δ^	1.41±0.11^†#^	1.52±0.12^†Δ^	1.53±0.14^†Δ^

Abbreviations: EF, %, ejection fraction; FS, %, percent fractional shortening; IVSd, interventricular septum thickness in end-diastole; LVEDD, left ventricle end-diastolic diameter; LVESD, left ventricle end-systolic diameter; LVmass, left ventricular mass

Sham: sham group; AMI: AMI group; MSCs: AMI+MSCs group; MSCs-GFP: AMI+MSCs-GFP group; MSCs-PKC*ɛ*: AMI+MSCs-PKC*ɛ*-GFP group; AMD3100: AMI+MSCs-PKC*ɛ*-GFP+AMD3100 group; LY294002: AMI+MSCs-PKC*ɛ*-GFP+LY294002 group

*P<0.05 *versus* AMI group; ^†^*P*<0.01 *versus* AMI group; ^‡^*P*<0.05 *versus* MSCs-GFP group; ^#^*P*<0.01 *versus* MSCs-GFP group; ^Δ^*P*<0.05 *versus* MSCs-PKC*ɛ* group.

## Abbreviations

PKC*ɛ*: protein kinase C *ɛ*
AMI: acute myocardial infarction
MSCs: mesenchymal stem cells
BMMSCs: bone marrow MSCs
SDF-1: stromal cell-derived factor 1
CXCR4: CXC chemokine receptor type 4
PI3K: phosphatidylinositol 3 kinase
AKT: protein kinase B
p-AKT: phosphorylated AKT
VEGF: vascular endothelial growth factor
bFGF: basic fibroblast growth factor
TGF*β*: transforming growth factor *β*
cTnI: cardiac troponin I
vWF: von Willebrand factor
SMA: smooth muscle actin
OD: optical density
GFP: green fluorescent protein
EGFP: enhanced GFP
LAD: left anterior descending branch
mRNA: messenger RNA
TUNEL: TdT-mediated dUTP nick end labeling
PCR: polymerase chain reaction
EF: ejection fraction
FS: percent fractional shortening
LVEDD: left ventricle end-diastolic diameter
LVESD: left ventricle end-systolic diameter
IVSd: interventricular septum thickness in end-diastole
LVmass: left ventricular mass
DAB: diaminobenzidine
TTC: 2,3,5,triphenyl-2H-tetrazolium chloride
LV: left ventricle
AAR: areas at risk
MAPK: mitogen-activated protein kinase
JNK: c-Jun N-terminal kinase
pJNK: phosphorylated JNK
CSCs: cardiac stem cells
