# Supplementary material for: Overexpression of protein kinase C ɛ improves retention and survival of transplanted mesenchymal stem cells in rat acute myocardial infarction
Source: Cell Death Dis. 2016 Jan 21;7(1):e2056–. doi: 10.1038/cddis.2015.417 (PMC4816190; doi:10.1038/cddis.2015.417)
Supplement: Supplementary Information [file cddis2015417x1.doc]

Table S1 Primer sequences

| Name | Sequence | Primer length (bp) | Tm (°C) | Product length (bp) |
| --- | --- | --- | --- | --- |
| PI3K-F | ATACCGTCAGCAGTTTGTCTC | 21 | 55.4 | 212 |
| PI3K-R | GCATCCAAGGGTCCAGTTAGT | 21 | 58.2 |  |
| PKCε-F | AAGGTGTTAGGCAAAGGCAG | 20 | 57.3 | 192 |
| PKCε-R | GCAGCAATAGAGTTGGGTTAG | 21 | 54.8 |  |
| AKT-F | TTTGGGAAGGTGATCCTGGTG | 21 | 62 | 184 |
| AKT-R | GGTCGTGGGTCTGGAATGAGT | 21 | 60.9 |  |
| CXCR4-F | TCTTCTTGACTGGCATAGTGG | 21 | 55.3 | 224 |
| CXCR4-R | GCTGTAAAGGTTGACGGTGTA | 21 | 55.6 |  |
| SDF-1F | CTGAATAGTGGCTCCCAAGGTT | 22 | 60.1 | 151 |
| SDF-1R | GTGGATCTCGCTCTTCCCTGAC | 22 | 62.5 |  |
| P38/MAPK-F | GGTTTTGGACTCGGATAAGA | 20 | 54.3 | 246 |
| P38/MAPK-R | GTGGGATGGACAGAACAGAAG | 21 | 56.6 |  |
| JNK-F | TTTGGGAAGGTGATCCTGGTG | 20 | 52.6 | 217 |
| JNK-R | TTTGGACGCATCTATCACC | 19 | 53.7 |  |
| bFGF-F | CGCACCCTATCCCTTCACAGC | 21 | 64.2 | 107 |
| bFGF-R | CAGCCTTCCACCCAAAGCAGT | 21 | 63.9 |  |
| TGFb-F | CAACAATTCCTGGCGTTACCT | 21 | 59.4 | 125 |
| TGFb-R | AGCCCTGTATTCCGTCTCCTT | 21 | 59.6 |  |
| VEGF F | CCCGACAGGGAAGACAAT | 18 | 50.4 | 131 |
| VEGF R | TCTGGAAGTGAGCCAACG | 18 | 51.2 |  |
| β-actin-F | GGAGATTACTGCCCTGGCTCCTAGC | 25 | 60.1 | 155 |
| β-actin-R | GGCCGGACTCATCGTACTCCTGCTT | 25 | 62 |  |

Table S2 Incubation conditions of the primary antibodies

| Primary antibody | Source | Dilution | Incubation condition |
| --- | --- | --- | --- |
| CD29 antibody | RabMAbs | 1:20 | 37°C 30 min |
| CD34 antibody | Santa Cruz Biotechnology | 1:20 | 37°C 30 min |
| CD44 antibody | Santa Cruz Biotechnology | 1:20 | 37°C 30 min |
| CD45 antibody | Santa Cruz Biotechnology | 1:20 | 37°C 30 min |
| p-PKCε antibody | Santa Cruz Biotechnology | 1:100 | 4°C overnight |
| PKCε antibody | Santa Cruz Biotechnology | 1:100 | 4°C overnight |
| p-JNK antibody | Santa Cruz Biotechnology | 1:100 | 4°C overnight |
| JNK antibody | Santa Cruz Biotechnology | 1:100 | 4°C overnight |
| p-P38 antibody | Santa Cruz Biotechnology | 1:100 | 4°C overnight |
| P38 antibody | Santa Cruz Biotechnology | 1:100 | 4°C overnight |
| SDF-1 antibody | Santa Cruz Biotechnology | 1:100 | 4°C overnight |
| CXCR4 antibody | Santa Cruz Biotechnology | 1:100 | 4°C overnight |
| PI3K antibody | Santa Cruz Biotechnology | 1:100 | 4°C overnight |
| p-AKT antibody | Santa Cruz Biotechnology | 1:100 | 4°C overnight |
| AKT antibody | Santa Cruz Biotechnology | 1:100 | 4°C overnight |
| TGFβ antibody | Santa Cruz Biotechnology | 1:100 | 4°C overnight |
| VEGF antibody | Bioss | 1:1000 | 4°C overnight |
| bFGF antibody | Santa Cruz Biotechnology | 1:100 | 4°C overnight |

Table S3 Incubation conditions of the secondary antibodies

| Primary antibody | Secondary antibody | Dilution | Incubation condition |
| --- | --- | --- | --- |
| CD29 antibody | FITC-labeled anti-rabbit secondary antibody | 1:20 | room temperature, away from light 30 min |
| CD34 antibody | FITC-labeled anti-mouse secondary antibody | 1:20 | room temperature, away from light 30 min |
| CD44 antibody | FITC-labeled anti-mouse secondary antibody | 1:20 | room temperature, away from light 30 min |
| CD45 antibody | FITC-labeled anti-mouse secondary antibody | 1:20 | room temperature, away from light 30 min |
| p-PKCε antibody | Goat anti-mouse IgG-HRP | 1:5000 | 37°C 45 min |
| PKCε antibody | Goat anti-mouse IgG-HRP | 1:5000 | 37°C 45 min |
| p-JNK antibody | Goat anti-mouse IgG-HRP | 1:5000 | 37°C 45 min |
| JNK antibody | Goat anti-mouse IgG-HRP | 1:5000 | 37°C 45 min |
| p-P38 antibody | Goat anti-rabbit IgG-HRP | 1:5000 | 37°C 45 min |
| P38 antibody | Goat anti-rabbit IgG-HRP | 1:5000 | 37°C 45 min |
| SDF-1 antibody | Goat anti-rabbit IgG-HRP | 1:5000 | 37°C 45 min |
| CXCR4 antibody | Goat anti-rabbit IgG-HRP | 1:5000 | 37°C 45 min |
| PI3K antibody | Goat anti-rabbit IgG-HRP | 1:5000 | 37°C 45 min |
| p-AKT antibody | Goat anti-rabbit IgG-HRP | 1:5000 | 37°C 45 min |
| AKT antibody | Goat anti-rabbit IgG-HRP | 1:5000 | 37°C 45 min |
| TGFβ antibody | Goat anti-rabbit IgG-HRP | 1:5000 | 37°C 45 min |
| VEGF antibody | Goat anti-rabbit IgG-HRP | 1:5000 | 37°C 45 min |
| bFGF antibody | Goat anti-mouse IgG-HRP | 1:5000 | 37°C 45 min |

Table S4 Antibodies for immunofluorescence

| Antibody | Dilution | Catalog number | Company | Incubation condition |
| --- | --- | --- | --- | --- |
| PKCε (mouse) | 1:50 | sc-1681 | Santa Cruz Biotechnology | 4°C overnight |
| cTnI (Rabbit) | 1:50 | sc-15368 | Santa Cruz Biotechnology | 4°C overnight |
| vWF (Rabbit) | 1:50 | sc-14014 | Santa Cruz Biotechnology | 4°C overnight |
| SMA (Rabbit) | 1:50 | ab108424 | Abcam | 4°C overnight |
| Cy3 labeled goat anti-mouse secondary antibody | 1:200 | A0521 | Beyotime | Room temperature 90 min |
| Alexa Fluor 647 labeled goat anti-rabbit secondary antibody | 1:200 | A0468 | Beyotime | Room temperature 90 min |

Table S5 TUNEL

| Assay kit | Catalog number | Company |
| --- | --- | --- |
| In Situ Cell Death Detection Kit | 11684817910 | Roche |

Table S6 Immunohistochemistry

| Antibody | Dilution | Catalog number | Company | Incubation condition |
| --- | --- | --- | --- | --- |
| Factor VIII | 1:100 | bs-2974R | Bioss | 4°C overnight |
| Biotin labeled goat anti-rabbit secondary antibody | 1:200 | A0277 | Beyotime | 37°C 30 min |


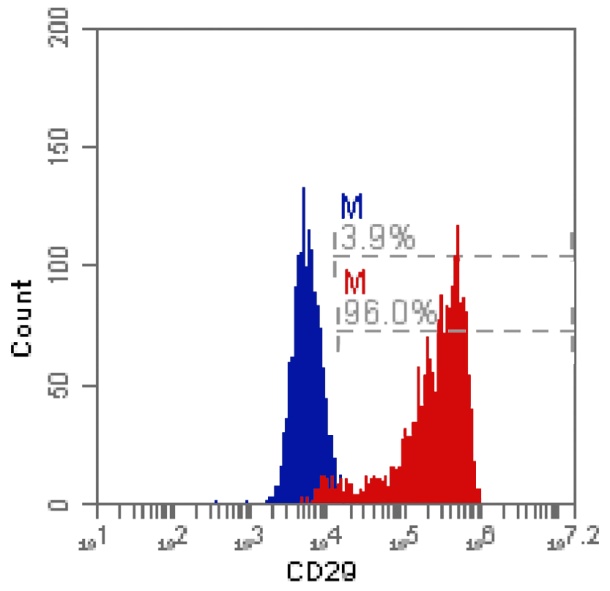

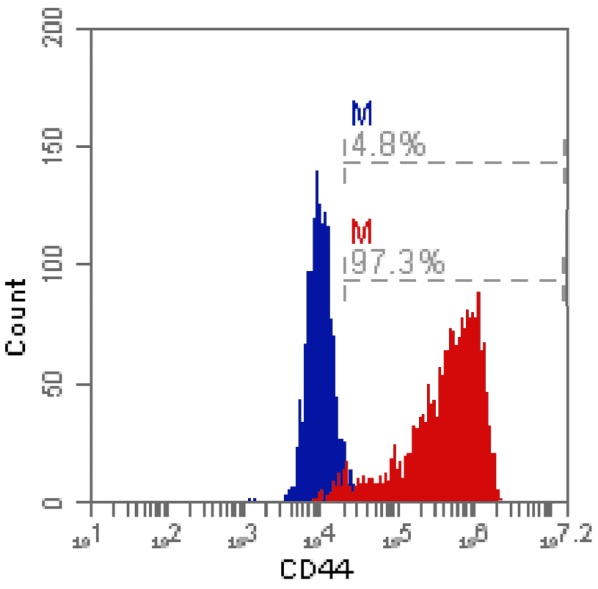

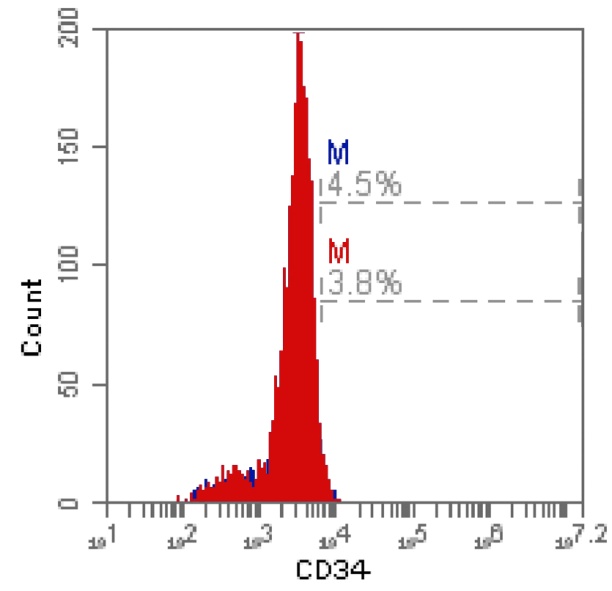

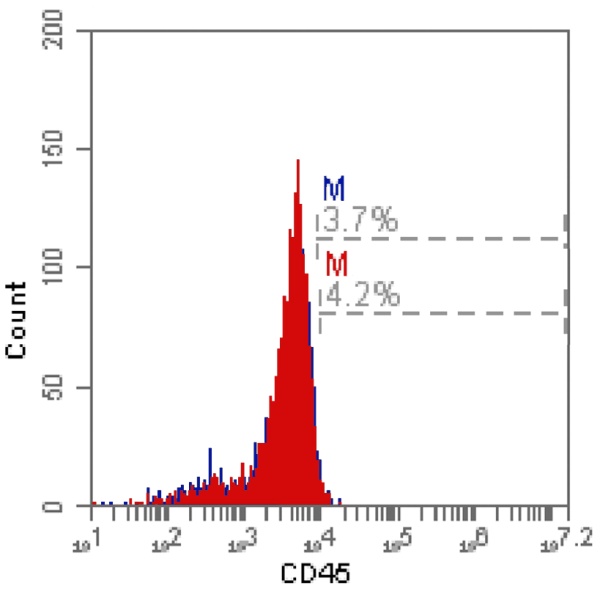


**CD29**

**CD45**

**CD44**

**CD34**

Figure S1

Figure S1. The phenotypic nature of rat BMMSCs. The red histograms show the fluorescence intensity of BMMSCs with antibody by flow cytometry (n=3); the blue histograms represents the isotype control. The phenotype of BMMSCs was shown to be positive for CD29, CD44 and negative for CD34, CD45.


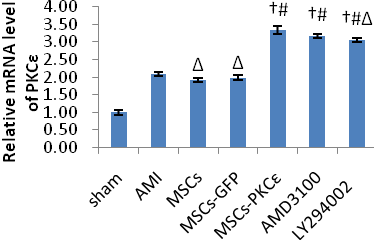

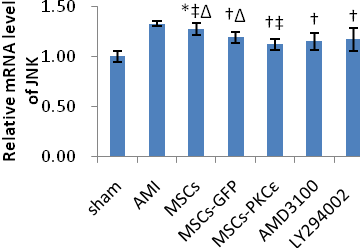


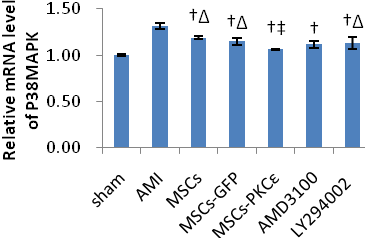

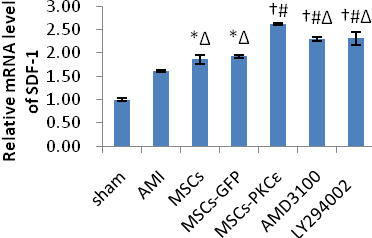


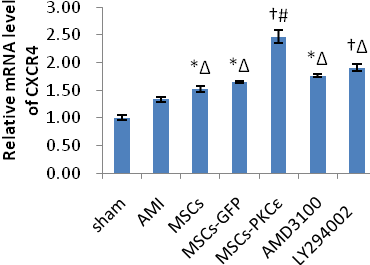

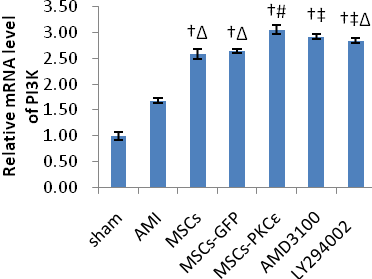


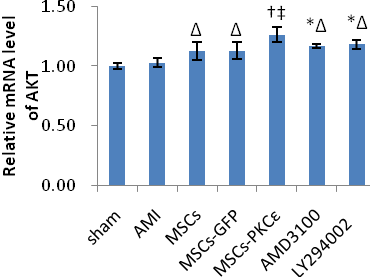
 Figure S2

Figure S2. mRNA of principal signal proteins in PKCε signaling, SDF-1/CXCR4 axis and PI3K/AKT pathway in each group 1 d after transplantation. Bar graphs show relative mRNA of PKCε, JNK, P38MAPK, SDF-1, CXCR4, PI3K and AKT, and β-actin is an internal reference. Data are means ± SD for five different experiments (n=6).

sham: sham group; AMI: AMI group; MSCs: AMI+MSCs group; MSCs-GFP: AMI+MSCs-GFP group; MSCs-PKCε: AMI+MSCs-PKCε-GFP group; AMD3100: AMI+MSCs-PKCε-GFP+AMD3100 group; LY294002: AMI+MSCs-PKCε-GFP+LY294002 group; * *P*< 0.05 *vs*. AMI group; † *P*< 0.01*vs.*AMI group; ‡ *P*< 0.05 *vs.* MSCs-GFP group; # *P*< 0.01 *vs.* MSCs-GFP group; Δ *P*< 0.05 *vs.* MSCs-PKCε group;


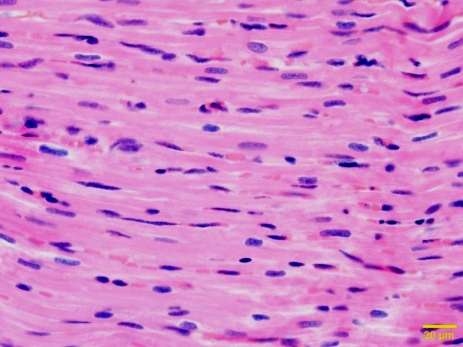

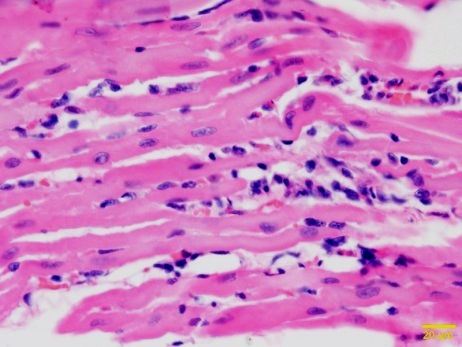

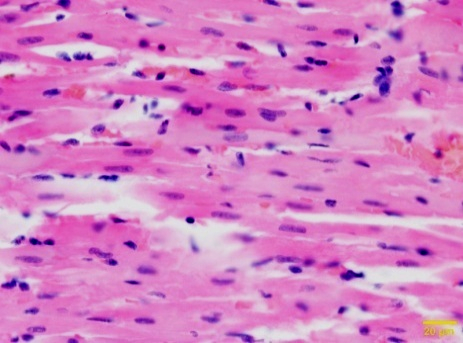


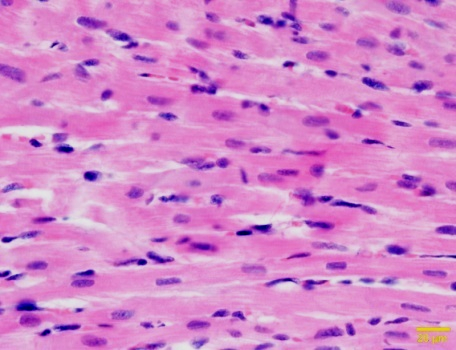

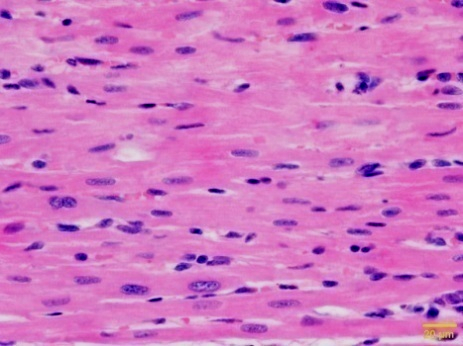

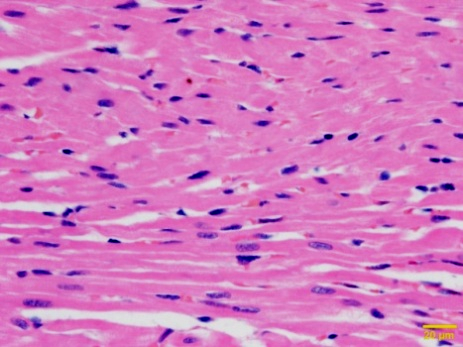


**Sham**

**AMI**

**MSCs**

**LY294002**

**MSCs-GFP**

**AMD3100**

**MSCs-PKCε**


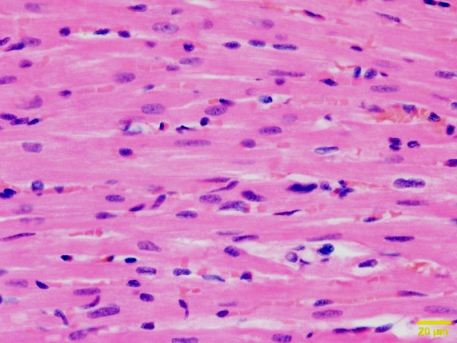


Figure S3A


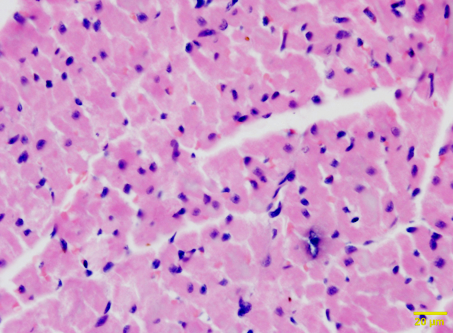

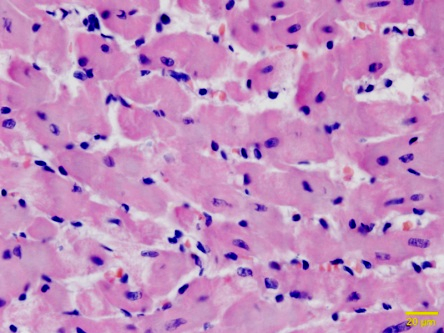

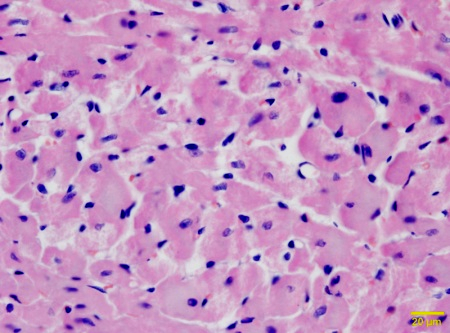


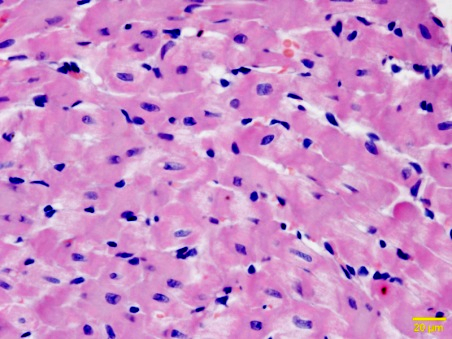

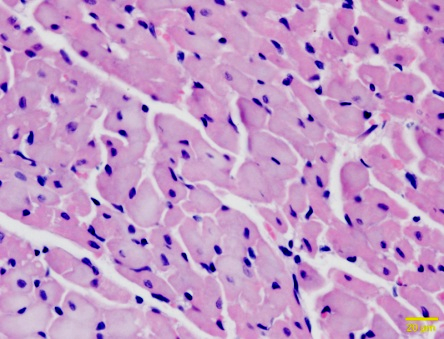

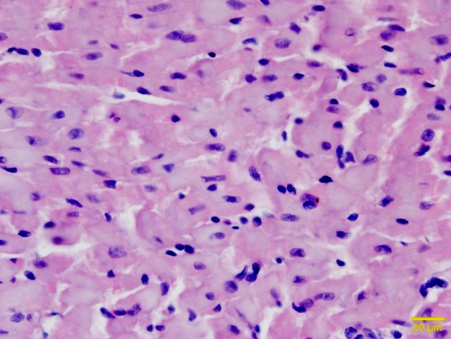


**MSCs-PKCε**

**MSCs**

**Sham**

**AMI**

**MSCs-GFP**

**AMD3100**

**LY294002**


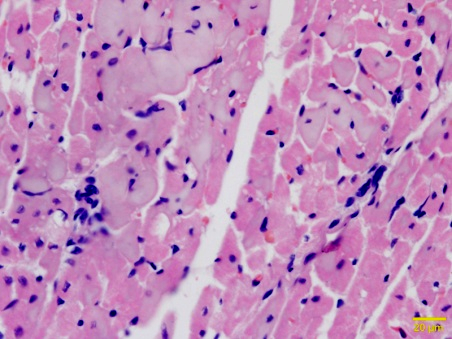

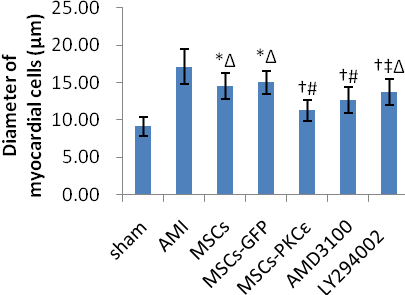


Figure S3B

Figure S3. Cardiac histology for each group 4 weeks after transplantation. Figure S3A: Cardiac histological longitudinal sections stained with hematoxylin and eosin (H&E) (600×) in each group. Figure S3B: Cardiac histological cross sections stained with H&E (600×) in each group. Bar graph shows diameters of myocardial cells per group. Data are means ± SD for three different experiments (n=4).

sham: sham group; AMI: AMI group; MSCs: AMI+MSCs group; MSCs-GFP: AMI+MSCs-GFP group; MSCs-PKCε: AMI+MSCs-PKCε-GFP group; AMD3100: AMI+MSCs-PKCε-GFP+AMD3100 group; LY294002: AMI+MSCs-PKCε-GFP+LY294002 group; * *P*< 0.05 *vs*. AMI group; † *P*< 0.01*vs.* AMI group; ‡ *P*< 0.05 *vs.* MSCs-GFP group; # *P*< 0.01 *vs.* MSCs-GFP group; Δ *P*< 0.05 *vs.* MSCs-PKCε group;


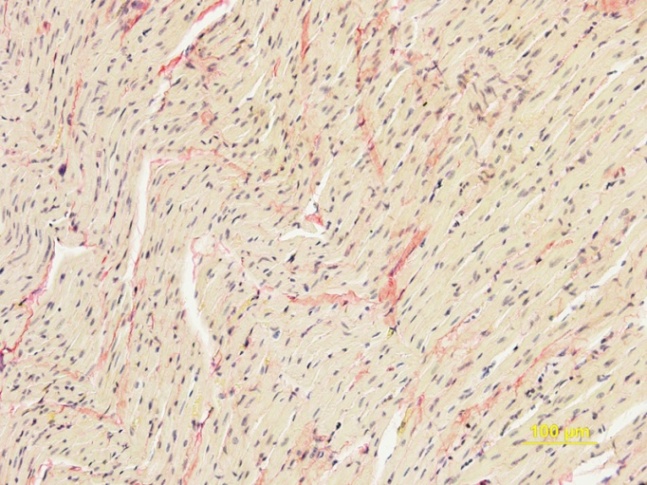

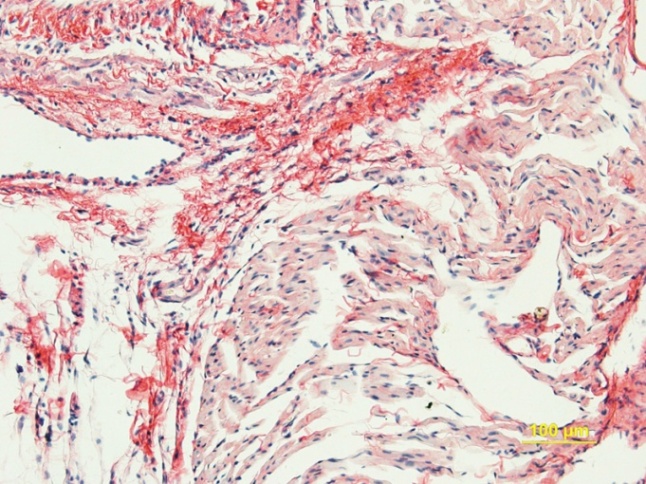


Infarct size (%)

**MSCs-GFP**

**Sham**

**MSCs**

**AMI**


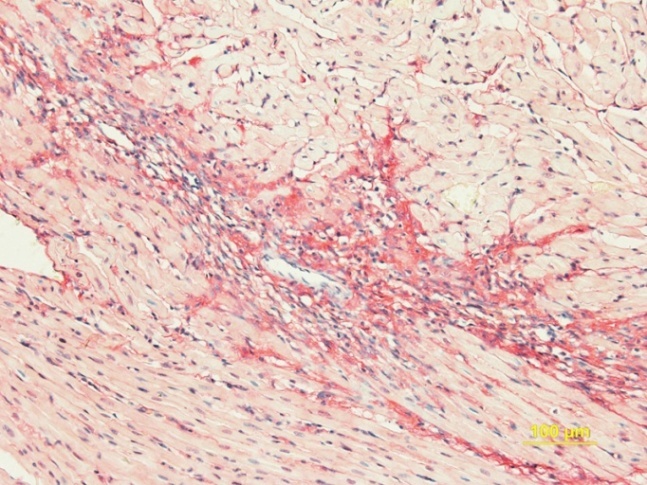

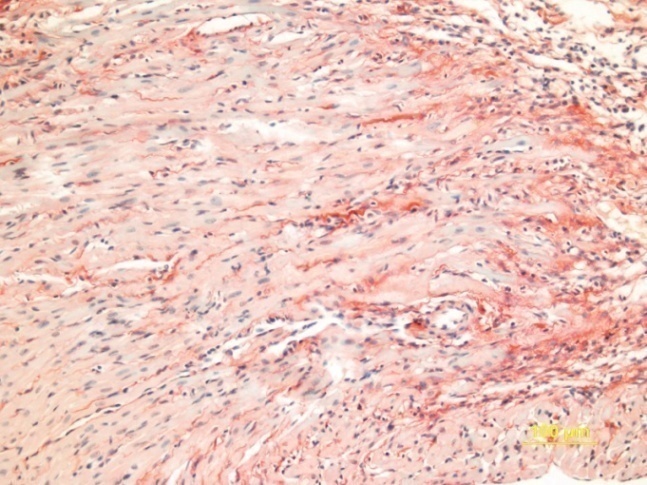


**MSCs-PKCε**

**AMD3100**


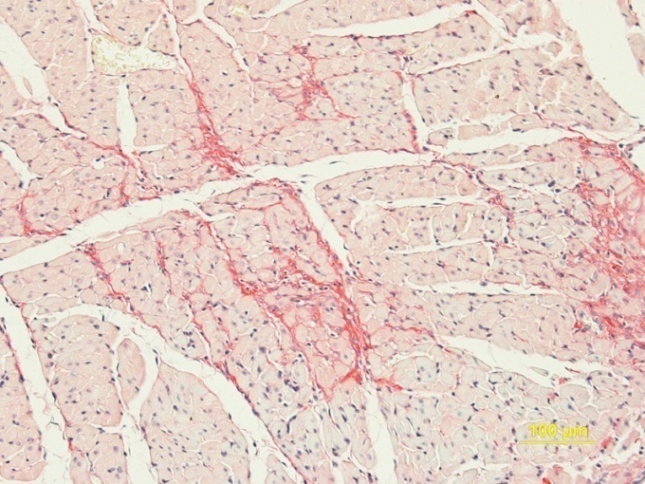

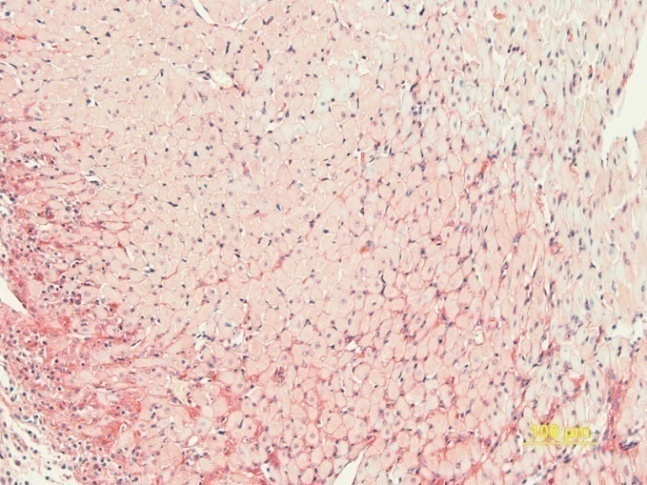


**LY294002**


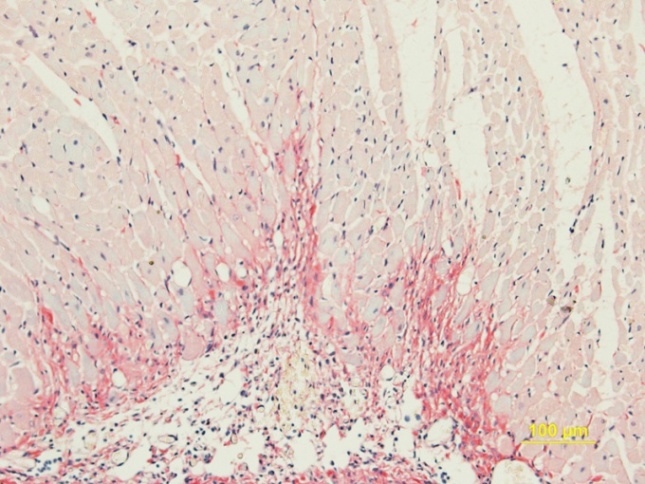

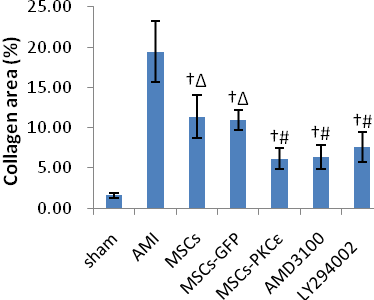


Figure S4

Figure S4. Cardiac fibrotic analysis for each group 4 weeks after transplantation. Cardiac histological sections stained with picro sirius red (200×) in each group are shown. Bar graph shows myocardial interstitial collagen area percent [(collagen area/total area) × 100%] in each group. Data are means ± SD for three different experiments (n=4).

sham: sham group; AMI: AMI group; MSCs: AMI+MSCs group; MSCs-GFP: AMI+MSCs-GFP group; MSCs-PKCε: AMI+MSCs-PKCε-GFP group; AMD3100: AMI+MSCs-PKCε-GFP+AMD3100 group; LY294002: AMI+MSCs-PKCε-GFP+LY294002 group; * *P*< 0.05 *vs*. AMI group; † *P*< 0.01*vs.*AMI group; ‡ *P*< 0.05 *vs.* MSCs-GFP group; # *P*< 0.01 *vs.* MSCs-GFP group; Δ *P*< 0.05 *vs.* MSCs-PKCε group;


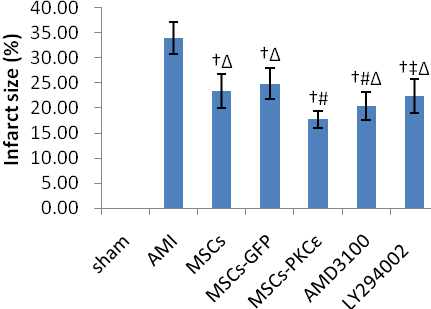


Figure S5

Figure S5. Infarct size for each group 4 weeks after transplantation. Bar graph shows infarction size measured by TTC in each group. Data are means ± SD for three different experiments (n=4).

sham: sham group; AMI: AMI group; MSCs: AMI+MSCs group; MSCs-GFP: AMI+MSCs-GFP group; MSCs-PKCε: AMI+MSCs-PKCε-GFP group; AMD3100: AMI+MSCs-PKCε-GFP+AMD3100 group; LY294002: AMI+MSCs-PKCε-GFP+LY294002 group; * *P*< 0.05 *vs*. AMI group; † *P*< 0.01 *vs.* AMI group; ‡ *P*< 0.05 *vs.* MSCs-GFP group; # *P*< 0.01 *vs.* MSCs-GFP group; Δ *P*< 0.05 *vs.* MSCs-PKCε group;


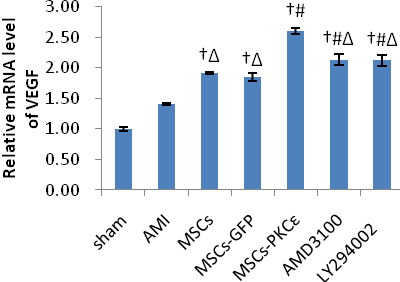

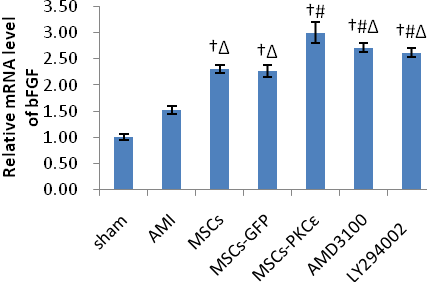


Infarct size (%)


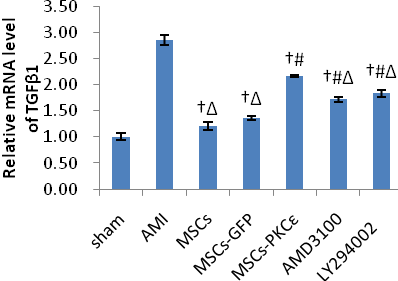


Figure S6

Figure S6. mRNA of VEGF, bFGF and TGFβ1 in each group 4 weeks after transplantation. Bar graphs show relative mRNA of VEGF, bFGF and TGFβ, and β-actin is an internal reference. Data are means ± SD for five different experiments (n=6).

sham: sham group; AMI: AMI group; MSCs: AMI+MSCs group; MSCs-GFP: AMI+MSCs-GFP group; MSCs-PKCε: AMI+MSCs-PKCε-GFP group; AMD3100: AMI+MSCs-PKCε-GFP+AMD3100 group; LY294002: AMI+MSCs-PKCε-GFP+LY294002 group; * *P*< 0.05 *vs*. AMI group; † *P*< 0.01*vs.*AMI group; ‡ *P*< 0.05 *vs.* MSCs-GFP group; # *P*< 0.01 *vs.* MSCs-GFP group; Δ *P*< 0.05 *vs.* MSCs-PKCε group.
